# Brain‐wide mapping of efferent projections of glutamatergic (Onecut3^+^) neurons in the lateral mouse hypothalamus

**DOI:** 10.1111/apha.13973

**Published:** 2023-04-25

**Authors:** Maja Zupančič, Evgenii Tretiakov, Zoltán Máté, Ferenc Erdélyi, Gábor Szabó, Frédéric Clotman, Tomas Hökfelt, Tibor Harkany, Erik Keimpema

**Affiliations:** ^1^ Department of Molecular Neurosciences, Center for Brain Research Medical University of Vienna Vienna Austria; ^2^ Institute of Experimental Medicine, Hungarian Academy of Sciences Budapest Hungary; ^3^ Animal Molecular and Cellular Biology Group, Louvain Institute of Biomolecular Science and Technology Université Catholique de Louvain Louvain‐la‐Neuve Belgium; ^4^ Department of Neuroscience, Biomedicum 7D Karolinska Institutet Solna Sweden

**Keywords:** development, reinforcer circuit, transcription factor, transgenic mouse model

## Abstract

**Aim:**

This study mapped the spatiotemporal positions and connectivity of *Onecut3* ^+^ neuronal populations in the developing and adult mouse brain.

**Methods:**

We generated fluorescent reporter mice to chart *Onecut3* ^+^ neurons for brain‐wide analysis. Moreover, we crossed *Onecut3*‐iCre and *Mapt*‐mGFP (Tau‐mGFP) mice to visualize axonal projections. A dual *Cre/Flp*‐dependent AAV construct in *Onecut3*‐iCre cross‐bred with *Slc17a6*‐FLPo mice was used in an intersectional strategy to map the connectivity of glutamatergic lateral hypothalamic neurons in the adult mouse.

**Results:**

We first found that *Onecut3* marks a hitherto undescribed *Slc17a6* ^+^/*Vglut2* ^+^ neuronal cohort in the lateral hypothalamus, with the majority expressing thyrotropin‐releasing hormone. In the adult, *Onecut3* ^+^/*Vglut2* ^+^ neurons of the lateral hypothalamus had both intra‐ and extrahypothalamic efferents, particularly to the septal complex and habenula, where they targeted other cohorts of *Onecut3* ^+^ neurons and additionally to the neocortex and hippocampus. This arrangement suggests that intrinsic reinforcement loops could exist for *Onecut3* ^+^ neurons to coordinate their activity along the brain's midline axis.

**Conclusion:**

We present both a toolbox to manipulate novel subtypes of hypothalamic neurons and an anatomical arrangement by which extrahypothalamic targets can be simultaneously entrained.

## 1 INTRODUCTION

The ever‐increasing pace of single‐cell RNA‐seq to recognize cellular diversity in the brain promotes the association of transcription factors (TFs) as identity marks to neuronal subtypes.^1^, ^2^, ^3^, ^4^, ^5^, ^6^ While this approach has unparallele d intrinsic power, neuronal populations that are placed outside conventional locations (be these layers, nuclei, or brain areas) are often overlooked. Our recent efforts to chart hypothalamic neuronal subtypes^7^, ^8^ led us to recognize the TF family containing a single CUT domain and a distinct homeodomain (*Onecut*)^9^ as a priority label for GABA/tyrosine hydroxylase^+^ neurons at the hypothalamic midline. *Onecut* TFs cascade from *Onecut1/2* expressed in progenitors to *Onecut3*, which labels postmitotic neurons in both the fetal and adult brains.^1^, ^2^ Nevertheless, the distribution of *Onecut3* ^+^ neurons along the rostrocaudal axis of the brain remains unaccounted for. Likewise, it is unclear if non‐GABA neurons could also express *Onecut3*, a concept that accords with the *Ascl1* origin of *Onecut3* ^*+*^ neurons^1^, ^10^; noting that hypothalamic *Ascl1* ^+^ progenitors can generate both GABA and glutamate neurons during cascading proliferation events.^10^

A major challenge in neurobiology is that the pace of the development of cellular and mouse models is inferior to that of advances in single‐cell RNA‐seq, as well as to spatial transcriptomics. This caveat is particularly relevant for the faithful manipulation of TFs. An efficient way to generate transgenic mice is the use of bacterial artificial chromosomes (BAC), which have large carrying capacity (hundreds of kilobases), for the efficient integration of promoter and regulatory elements of specified genes together with either transgenes (e.g., *Cre* recombinase, flippases [FLP]) or reporter molecules (e.g., DsRed, mCherry) into the host chromatin by homologous recombination.^11^ Here, we generated *Onecut3*‐mCherry and *Onecut3*‐iCre mouse lines and used these to map the brain‐wide distribution of *Onecut3* ^+^ neurons, and their connectivity, in the developing and adult nervous systems. We then combined these transgenic tools with immunohistochemistry, in situ hybridization, and high‐resolution tissue‐wide imaging to reveal the temporal trajectory of how *Onecut3* ^+^ neurons populate brain areas from mid‐gestation on in mouse, including the septal complex, hypothalamus, habenula, midbrain, and hindbrain. While *Onecut3* ^+^ nuclei form a quasi‐continuum along the midline, we conspicuously found *Onecut3* ^+^ neurons that populate the lateral hypothalamus. We then combined intersectional genetics (*Onecut3*‐iCre; *Slc17a6*‐FLPo) with an adeno‐associated viral (AAV) approach^12^ to map efferent projections of this *Onecut3* ^+^ subgroup, many of which containing thyrotropin‐releasing hormone (TRH). We found *Onecut3* ^+^ neurons of the lateral hypothalamus to predominantly target *Onecut3* ^+^ neurons in brain areas endowed with TRH receptors (*Trhr*), with their presynapses containing VGLUT2. Thus, we suggest that *Onecut3* ^+^ excitatory neurons of the lateral hypothalamus are poised to coordinate the activity of both intra‐ and extrahypothalamic *Onecut3* ^+^ neuronal pools.

## 2 RESULTS

### 2.1 Methodological considerations

**FIGURE 1:**
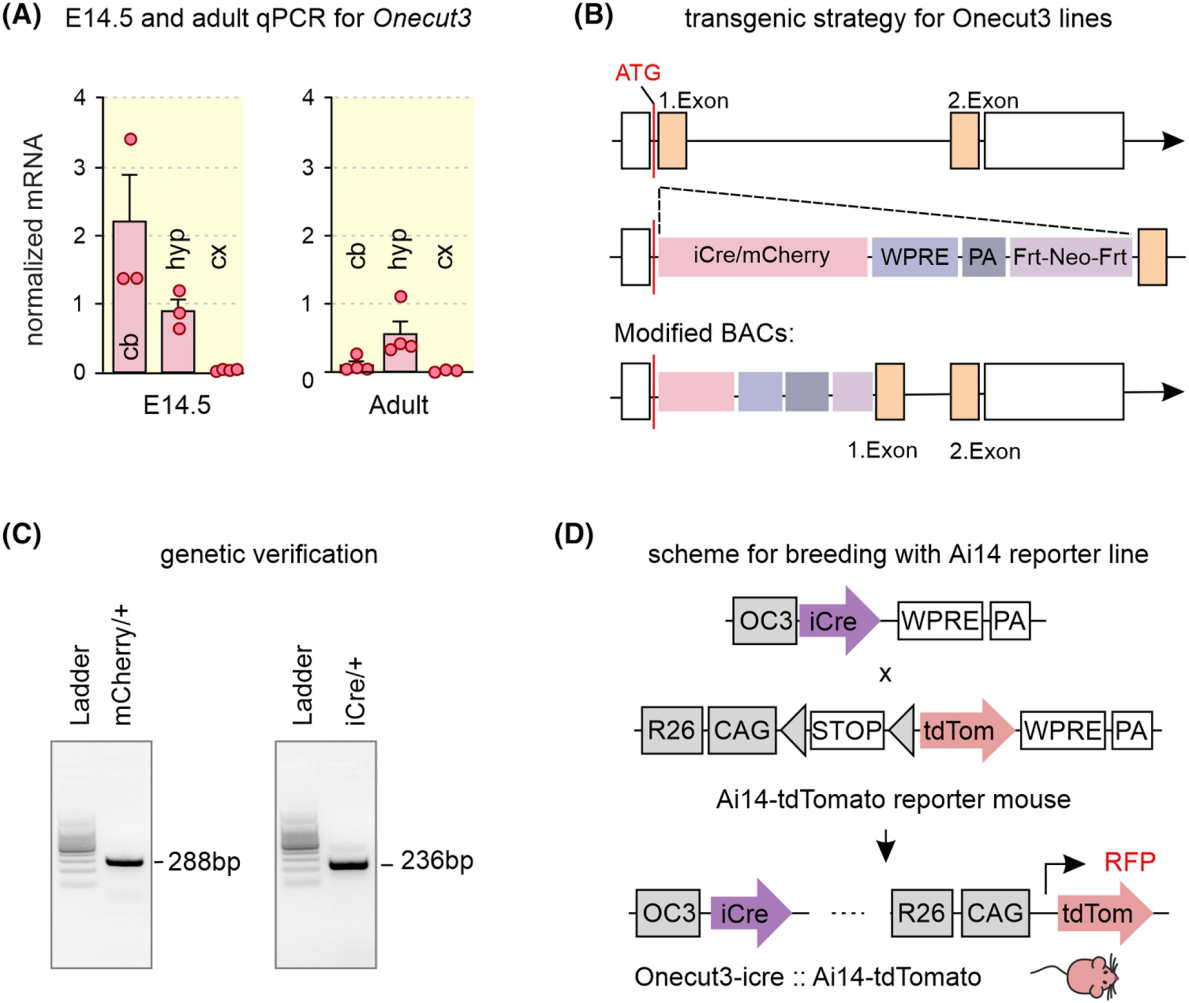
Generation of transgenic animal lines. (A) Normalized expression of *Onecut3* mRNA in the E14.5 and adult cerebellum (cb), hypothalamus (hyp) and cerebral cortex (cx). (B) Schematic design of the BAC transgene strategy used to generate mice carrying *Onecut3* constructs. (C) Standard genotyping used to confirm the presence of inserts for GFP, mCherry, or iCre transgenes. (D) Breeding schema for *Onecut3*‐iCre and Ai14 (R26‐tdTomato) mice. BAC, bacterial artificial chromosome; bp, base pair; cb, cerebellum; cx, cortex; GFP, green fluorescent protein; hyp, hypothalamus; OC3, *Onecut3*; PA, polyadenylation signal; WPRE, Woodchuck hepatitis virus posttranscriptional regulatory element. Data in (A) were expressed as means ± SD; solid circles correspond to individual data points.

Members of the *Onecut* family of TFs have so far been identified as critical for the development of hepatocytes, endocrine cells of the pancreas and the gastrointestinal tract, and the spinal cord.^13^, ^14^, ^15^, ^16^, ^17^, ^18^, ^19^, ^20^ Nevertheless, their localization in the brain remains incomplete particularly because of the lack of genetic tools to faithfully chart the distribution of neurons expressing *Onecut* paralogs, and to interrogate their connectivity to infer functions. We expect that generating tools to manipulate these TFs is of value particularly since the most subordinate member of the *Onecut* family, *Onecut3*, seems to be unconventional in the temporal and subregional regulation of its expression (Figure 1A), as single‐cell RNA‐seq suggested its mRNA being retained even in adulthood.^2^ Therefore, we used BAC‐based genetic engineering (Figure 1B, Tables 1 and 2) to generate mouse strains that carry either fluorescence reporters (mCherry) or iCre recombinase under regulatory elements of the *Onecut3* promoter and are stably integrated in the mouse genome (Figure 1B,C). (BAC)*Onecut3*‐mCherry mouse lines exhibited cellular fluorescence at levels that a mandatory amplification step by indirect fluorescence was not required for successful localization/mapping by laser‐scanning microscopy. Nevertheless, *Onecut3*‐iCre mice, when crossed with, e.g., the Ai14 reporter line (*Onecut3*‐iCre::Ai14) to express tdTomato (Figure 1D), had fluorescence intensities in excess of (BAC)*Onecut3*‐mCherry mice. Therefore, we only recognized a structure as a genuine *Onecut3* ^*+*^ locus if (i) it was labeled in both (BAC)*Onecut3*‐mCherry and *Onecut3*‐iCre::Ai14 mice, and (ii) also by indirect immunofluorescence using anti‐*Onecut3* antibodies that had been quality‐controlled in *Onecut3* ^−/−^ mice.^21^ Finally, *Onecut3*‐iCre mice allowed us to develop intersectional strategies to identify both the neurotransmitter identity and brain‐wide efferent maps of *Onecut3* ^+^ neurons localized at an unexpected location in the lateral hypothalamus.

**TABLE 1:** Animal lines.

Transgenic animal	Reference
BAC‐Onecut3‐mCherry (3, 7, 28)	This paper
BAC‐Onecut3‐iCre (1, 3, 9, 18, 21, 23, 29, 33, 42)	This paper
B6.Cg‐Gt(ROSA)26Sortm14(CAG‐tdTomato)Hze/J (Ai14‐tdTomato)	Jackson Laboratory #007914
B6;129P2‐Mapttm2Arbr/J (Tau‐mGFP)	Jackson Laboratory #021162
B6;129S‐Slc17a6tm1.1(flpo)Hze/J (Slc17a6‐FLPo)	Jackson Laboratory #030212

**TABLE 2:** Genotyping primers.

Transgenic animal	Genotyping primer pairs
*Onecut3*‐iCre	Forward: 5′‐AGATGCCAGGACATCAGGAACCTG‐3′ Reverse: 5′‐ ATCAGCCACACCAGACACAGAGATC‐3′
*Onecut3*‐mCherry	Forward: 5′‐AGGACGGCGAGTTCATCTAC‐3′ Reverse: 5′‐TGGTGTAGTCCTCGTTGTGG‐3′

### 2.2 *Onecut3* expression during fetal development

**FIGURE 2:**
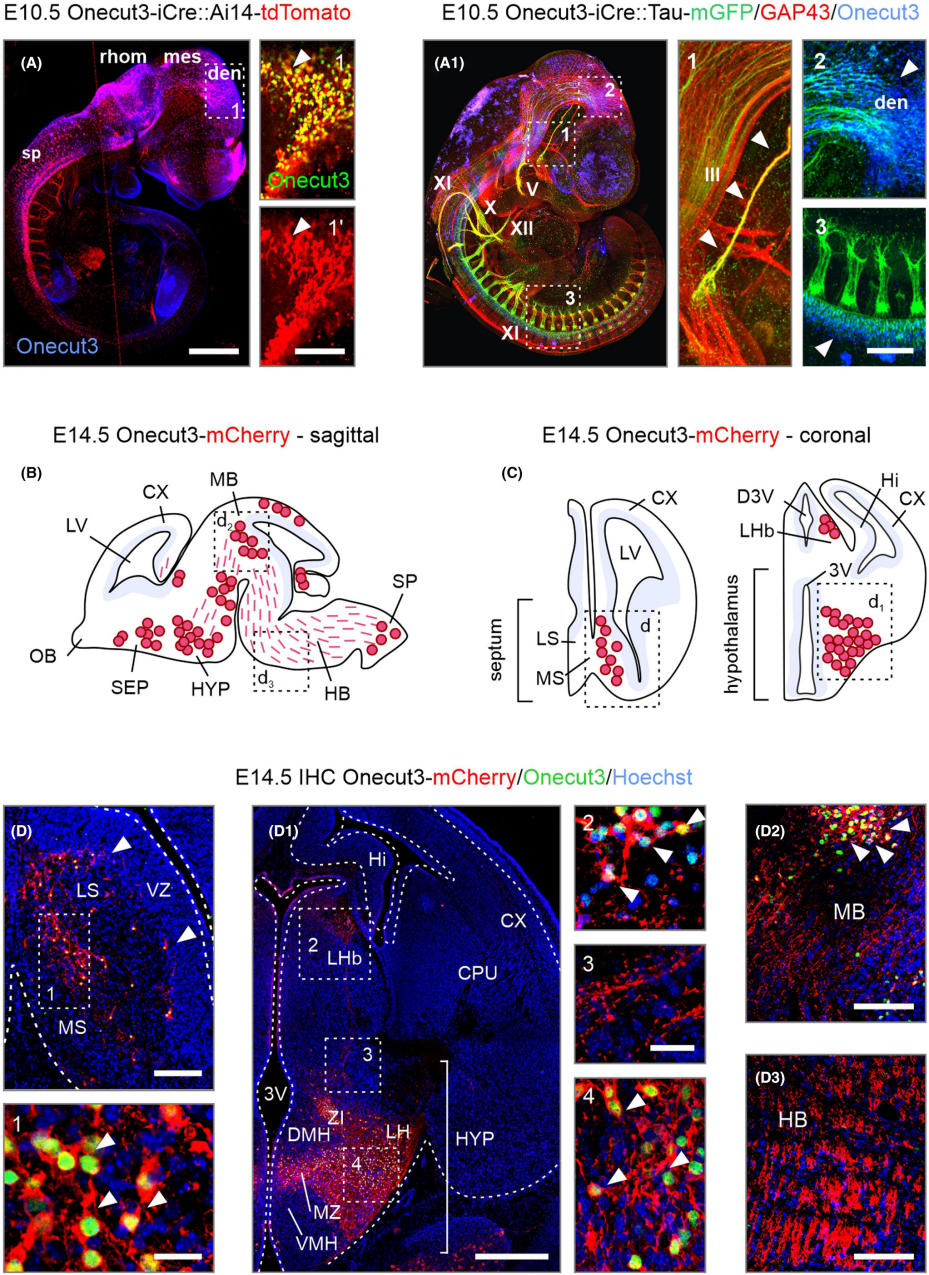
*Onecut3* ^+^ neurons and their projections in mouse embryos. (A) A chain of *Onecut3* ^+^ territories in the brain and along the spinal cord, including mCherry^+^ somata (arrowheads, A) and GFP^+^ axons (arrowheads, A_1_), were identified using *Onecut3*‐iCre mice. Insets in (A_1_) show the oculomotor nerve (1), corticospinal tract (2), and spinal segments (3). Sagittal (B) and coronal (C) brain maps on E14.5 used to schematically illustrate the distribution of mCherry^+^ cell bodies (red circles) and processes (dashed red lines). Histochemistry for Onecut3 (green) overlapped with mCherry (red, arrowheads), thus confirming faithful localization of the genetic signal. (D–D_3_). Onecut3^+^/mCherry^+^ perikarya were localized to the medial septum (D), hypothalamus (hyp) and habenula (Lhb; D_1_) midbrain (MB; D_2_) and hindbrain (D_3_). III,V, X, XI, XII, cranial nerves; 3V, 3rd ventricle; CX, cortex; den, diencephalon; HB, hindbrain; Hi, hippocampus; LV, lateral ventricle; MB, midbrain; mes, mesencephalon; MZ, mediolateral zone; OC3, Onecut3; OB, olfactory bulb; rhom, rhombencephalon; SEP, septum; sp, spinal cord; VZ, ventricular zone. Scale bars = 500 μm (A, B), 200 μm (D_1_), 100 μm (insets in A, D, D_2_, D_3_), 40 μm (insets in D, D_1_).

As conventional for many TFs that define neuronal identity, the *Onecut1* and *Onecut2* paralogs are expressed in hypothalamic progenitors in the rodent forebrain.^1^ In contrast, we found *Onecut3* in post‐mitotic cells that had exited the proliferative zones of the brain, alike in the spinal cord, (Figure 2A) by mid‐gestation in mouse. This finding was validated by combining mouse genetics and indirect histochemistry (Figure 2A), which ensured the authentic presence of tdTomato^+^ somata in the telencephalon, diencephalon, mesencephalon, rhombencephalon, and spinal cord. Besides localizing neuronal somata, we made an effort to reveal the extent, specific trajectories, and body‐wide targets of *Onecut3* ^+^ neurons of the nervous system. To this, we have crossed *Onecut3*‐iCre and *Mapt*‐(Tau)‐mGFP mice, the latter serving as an axonal reporter through its membrane‐bound GFP expression (Figure 2A_1_). Whole‐mount imaging, incorporating growth‐associated protein 43 (GAP43) to preferentially mark neurites and their growth cones during neuritogenesis,^22^, ^23^ demonstrated that GFP^+^ processes, likely axons, radiated through the diencephalon (Figure 2A_1_). In addition, we located Onecut3 protein in the spinal cord,^14^, ^15^, ^16^ most likely motorneurons and spinal ventral interneurons,^15^ with spinal nerves also positive for GFP (Figure 2A_1_). Finally, we localized GFP to the 3rd (oculomotor), 5th (trigeminal), 10th (vagus), 11th (accessory), and 12th (hypoglossal) nerves, indicating the presence of Onecut3 mainly in cranial nerve nuclei of neural crest origin.^24^ Confirming a previous report,^25^ *Onecut3* mRNA and protein overlapped with mCherry in retinal ganglion cells of the eye (Figure S1A).

We then used tissue clearing and 3D imaging by light‐sheet microscopy to produce a refined map of mCherry^+^ neuronal cell bodies in the forebrain at E14.5 (both sagittal and coronal views are shown; Figure 2B,C). This identified midline structures, wherein neurons primarily migrate from the wall of the 3rd ventricle: the medial septal area, hypothalamus, and midbrain. Brain areas in which the mCherry reporter overlapped with *Onecut3* mRNA, protein, or both, were only accepted as positively labeled (Figure 2D–D_3_; Figures S1B and S2A,B). The segregation of neuronal cohorts populating the prospective lateral vs. medial septal nuclei was noted as early as E14.5 (Figure 2D), together with the impending accumulation of *Onecut3* ^+^ neurons in the lateral habenula (Figure 2D_1_). The hypothalamus provided a curious case because the mCherry signal, including both perikaryal and processes, formed a continuous meshwork encompassing the periventricular nucleus (PeVN), mediolateral zone (MZ), and lateral hypothalamus (LH), and extending dorsally to the zona incerta (ZI), which partitions as a nucleus of the subthalamus.^1^, ^4^ A *Onecut3* ^+^ cell group was also found positioned behind the isthmic organizer in the dorsal midbrain, yet its identity was not further pursued in this study (Figure 2D_2_). In the hindbrain, bundles of axons were seen that reached the spinal cord (Figure 2D_3_).

### 2.3 *Onecut3* expression in adult brain

**FIGURE 3:**
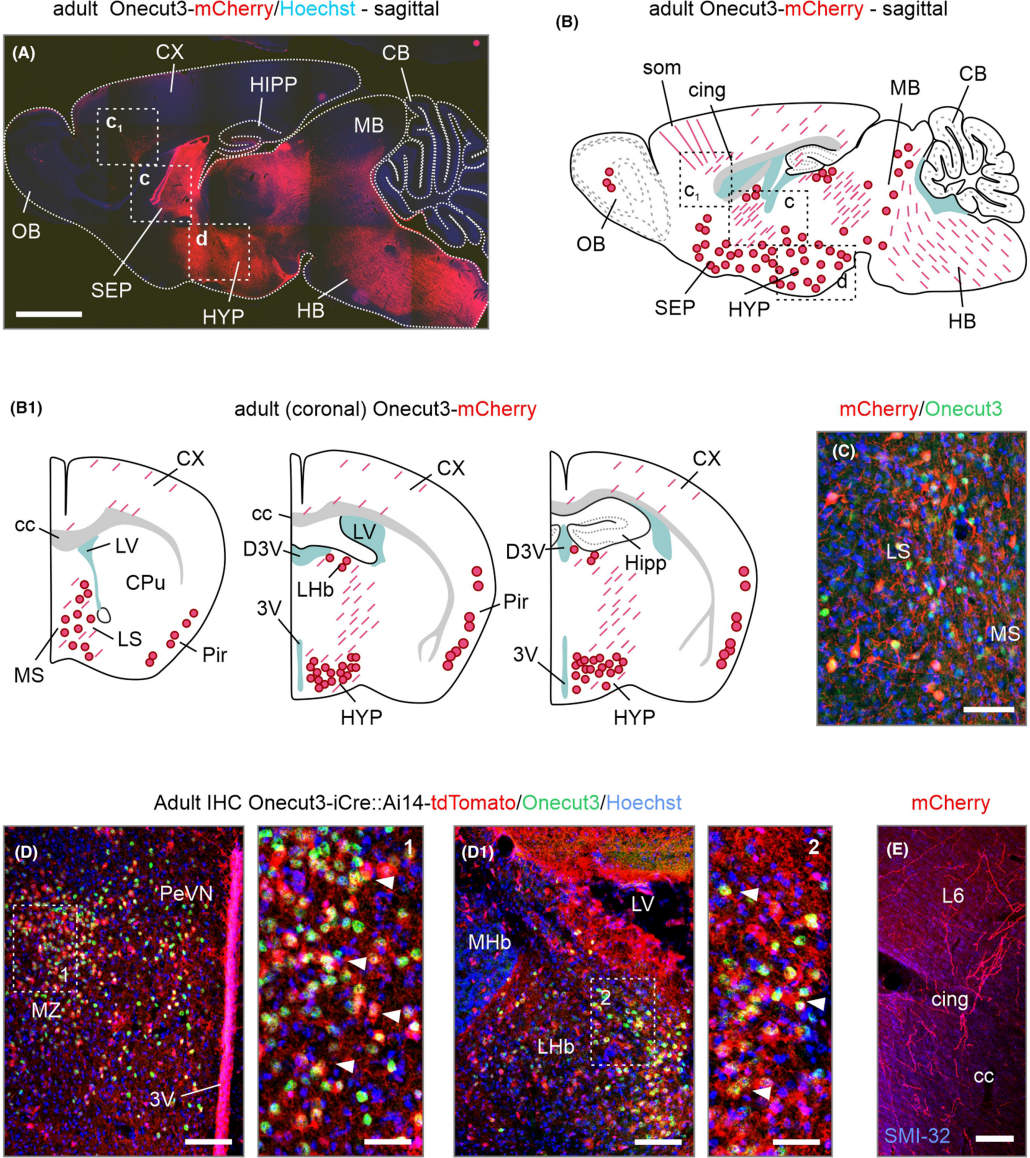
*Onecut3* ^+^ brain regions, and their mapping and in adulthood. (A) mCherry expression in a sagittal section encompassing midline structures in an adult *Onecut3*‐mCherry mouse brain. (B, B_1_) Schematic sagittal (B) and coronal (B_1_) depiction of mCherry^+^ cell bodies (solid red circles) and processes (dashed red lines). Note that the majority of mCherry^+^ neurons reside within the septal complex and hypothalamus. (C) Histochemistry for Onecut3 (green, *arrowheads*) in the septum of an adult *Onecut3*‐mCherry mouse. (D, D_1_) Concentration of tdTomato^+^ neurons and their processes in the periventricular hypothalamus and lateral habenula (LHb) of an adult *Onecut3*‐iCre::A14 mouse. (E) Fibers radiating from the cingulum into the somatomotor region. 3V, third ventricle; CB, cerebellum; cc, corpus callosum; cing; cingulum; CX, cortex; HB, hindbrain; HIPP, hippocampus; HYP, hypothalamus; L, layer; LS, lateral septum; LV, lateral ventricle; MB, midbrain; MHb, medial habenula; MS, medial septum; MZ, mediolateral zone; PeVN, periventricular nucleus; SEP, septum. Scale bars = 1 mm (A), 250 μm (E), 100 μm, (C), 50 μm (D, D_1_) 20 μm (insets, D).

Within the early postnatal brain, the pattern of mCherry^+^ neuronal structures recapitulated those identified at E14.5 (Figure 3A–B_1_; Figures S2A,B and S3A). When using the anatomical mapping tools as for embryonic brains, we found the co‐existence of mCherry and *Onecut3* protein in the adult lateral and medial septal nuclei (Figure 3B_1_,C). Within the hypothalamus, tdTomato^+^ neurons overlapped with *Onecut3* ^+^ protein and populated the anterior hypothalamic area, with their positions spread toward the wall of the 3rd ventricle, corresponding to the periventricular nucleus (PeVN; Figure 3D). Moreover, the MZ also harbored *Onecut3* ^+^ neurons (Figure 3D). In the lateral habenula (LHb), *Onecut3* ^+^ neurons concentrated in its magnocellular division (Figure 3B_1_,D_1_). In the medial habenula, a tdTomato^+^ cluster of neurons resided in the superior region; though we failed to detect *Onecut3* protein in this area. mCherry^+^ fiber labeling was found in the cingulum bundle, radiating toward layer 1 in the somatomotor area (Figure 3E). In addition, we found fine‐caliber fibers coursing mainly through layer 5/6 (its lamina distinguished through the density of both parvalbumin^+^ interneurons and SMI‐32^+^ pyramidal‐like cells, Figure S3B)^26^ throughout the sensory motor and visual cortices into the hippocampal CA1‐CA3 subfields (Figure S3B_1_,B_2_), while being excluded from the dentate gyrus (Figure S3B_3_). From a methodological standpoint, we find it important to emphasize that *Onecut3*‐Cre::Ai14 mice confirmed the cellular distribution maps we have charted by using (BAC)*Onecut3*‐mCherry mice, as shown for both the PeVN/MZ of the hypothalamus (Figure 3D, Figure S3B_5_), and LHb (Figure 3D_1_). Nevertheless, we caution that ectopic mCherry labeling (that is, no somatic co‐localization with *Onecut3* protein) occurred in the rostral migratory stream, olfactory bulb, and piriform cortex in (BAC)*Onecut3*‐mCherry mice, which are known for unwanted recombination also in some other (BAC) models (data not shown).^27^ For the *Onecut3*‐iCre::Ai14 line in particular, we found non‐complementary expression between tdTomato and *Onecut3* protein at some sites. These data could suggest developmentally regulated transient *Onecut3* expression, with Cre‐mediated recombination conferring “life‐time” tracing of these cell groups or ectopic recombination events. Therefore, (BAC)*Onecut3*‐mCherry mice are best suited for anatomical mapping studies of connectivity, while *Onecut3*‐iCre are useful for circuit reconstruction of novel cell groups through viral transduction so long as cellular targets are confirmed by a combination of transgenic and antibody‐based histochemical methods.

### 2.4 Brain‐wide circuit mapping using intersectional mouse genetics

**FIGURE 4:**
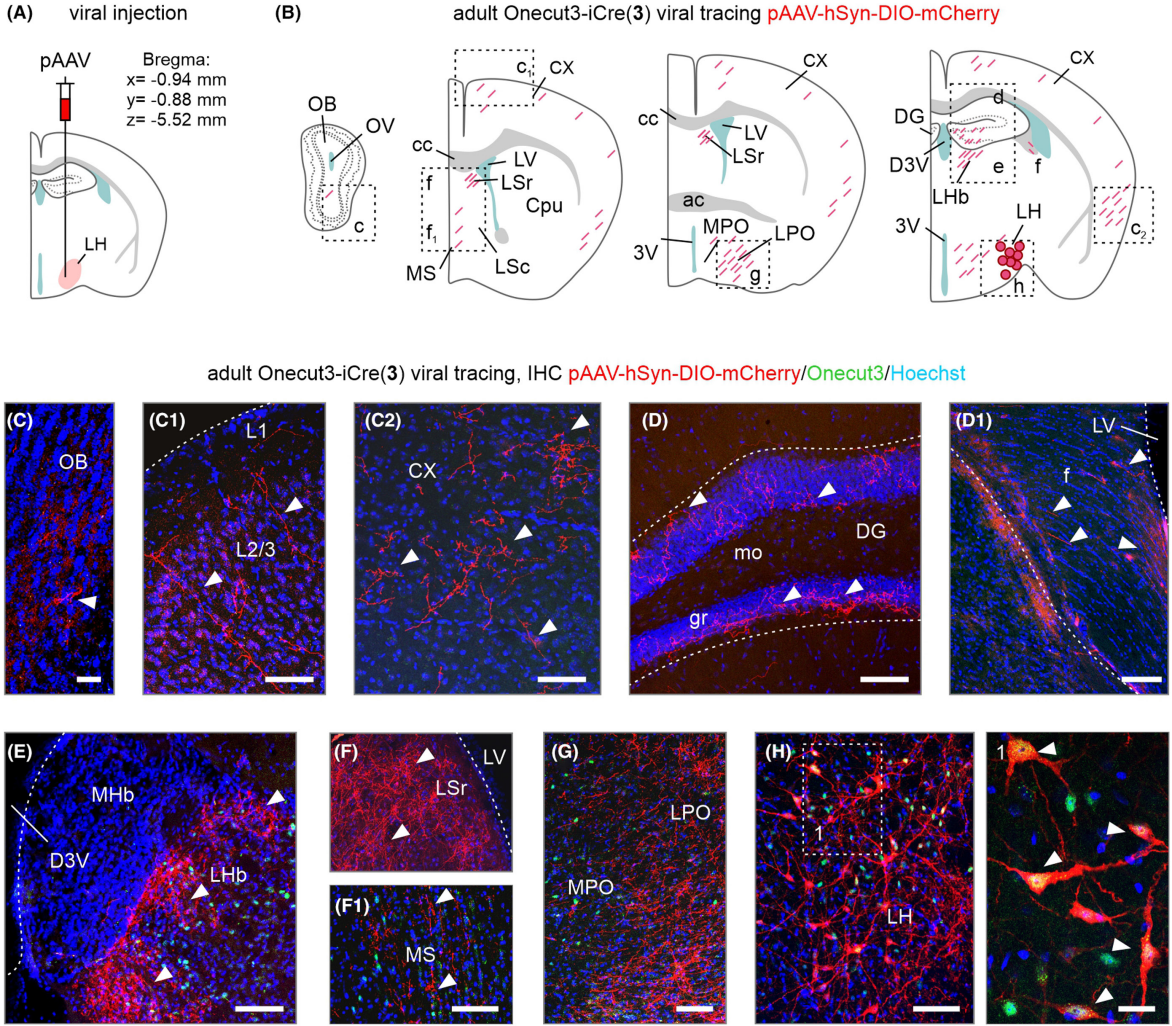
Indiscriminate viral transduction of *Onecut3* ^*+*^ neurons in the adult lateral hypothalamus. (A) Coordinates relative to bregma to target the lateral hypothalamic area. *Cre*‐dependent mCherry‐expressing virus particles (pAAV‐hSyn‐DIO‐mCherry) were injected to reveal efferent projections of *Onecut3* ^+^ neurons. (B–H) Brain‐wide mapping of mCherry^+^ axons originating in *Onecut3* ^+^ neurons of the lateral hypothalamus. Graphical rendering (B) and representative immunohistochemically images for the olfactory bulb (C), layer 2/3 of the somatosensory cortex (C_1_), ecto/perirhinal cortex (C_2_), the dentate gyrus (D), fimbria hippocampi (D_1_), the lateral habenula (E), lateral and medial septal nuclei (F, F_1_), the medial and lateral preoptic area (G) and the injection site with cell bodies (H). Arrowheads point to mCherry^+^ fibers. 3V, 3rd ventricle; ac, anterior commissure; Cpu, caudate putamen; cc, corpus callosum; CX, cortex; DG, dentate gyrus; fi, fimbria; gr, granular layer; LHb, lateral habenula; LSc, lateral septal nucleus caudal division; LSr, lateral septal nucleus rostral division; LPO, lateral preoptic area; LV, lateral ventricle; MHb, medial habenula; mo, molecular layer; MPO, medial preoptic area; MS, medial septum; OB, olfactory bulb. Scale bars = 500 μm (D), 250 μm (E, G), 50 μm (C, C_1_,C_2_, F, F_1_, H), 10 μm (H, inset; D_1_).

Next, we used our *Onecut3*‐iCre model to define the extent of both local and long‐range projections of these poorly understood lateral hypothalamic neurons. We first used a pAAV‐hSYN‐DIO‐mCherry‐driven adeno‐associated virus (AAV) approach, harboring mCherry under the neuron‐specific human synapsin promoter, in the lateral hypothalamus of adult *Onecut3*‐iCre mice (AP = −0.94, L = −0.88, DV = −5.52 mm, relative to bregma and dura as appropriate; *n* = 6, males and females; Figure 4A). After 3 weeks, mCherry signal was restricted to neurons in the lateral hypothalamus, indicating successful targeting (Figure 4B,H). Histochemistry for Onecut3 showed that these cells were correctly visualized by mCherry (Figure 4H), and had medium‐to‐thick processes, likely dendrites. Reminiscent to data from (BAC)*Onecut3*‐mCherry mice, sparse fiber labeling was detected in the olfactory bulb (Figure 4C), layers 1 and 2/3 of the somatosensory cortex (Figure 4C_1_), as well as the perirhinal and entorhinal areas (Figure 4C_2_). We also found fine‐caliber mCherry^+^ processes at the outer border of the granule cell layer of the hippocampal dentate gyrus (Figure 4D), which likely invaded through the fimbria hippocampi (Figure 4D_1_). Finally, we mapped long‐range mCherry^+^ fibers in the LHb (Figure 4E), the lateral and medial septal nuclei (Figure 4F,F_1_), as well as the tuberal region and lateral and medial preoptic area (Figure 4G), resolving a circumscribed neurocircuit with primary terminal fields dominating in brain regions that themselves harbor *Onecut3* ^+^ neurons (such as the LHb and septum; Figure 3). Given the anatomical position of the ZI, we in some cases virally labeled its *Onecut3* ^+^ neurons, with their efferent projections mapped separately (Figure S4). Considering that the presence of GABA/*Onecut3* ^+^ neurons^1^ in the lateral hypothalamaus cannot be excluded per se, even if these cells predominate in the PeVN, we opted for an intersectional approach to strengthen our conclusions on brain‐wide efferents of *Slc17a6*/*Onecut3* ^+^ neurons.

**FIGURE 5:**
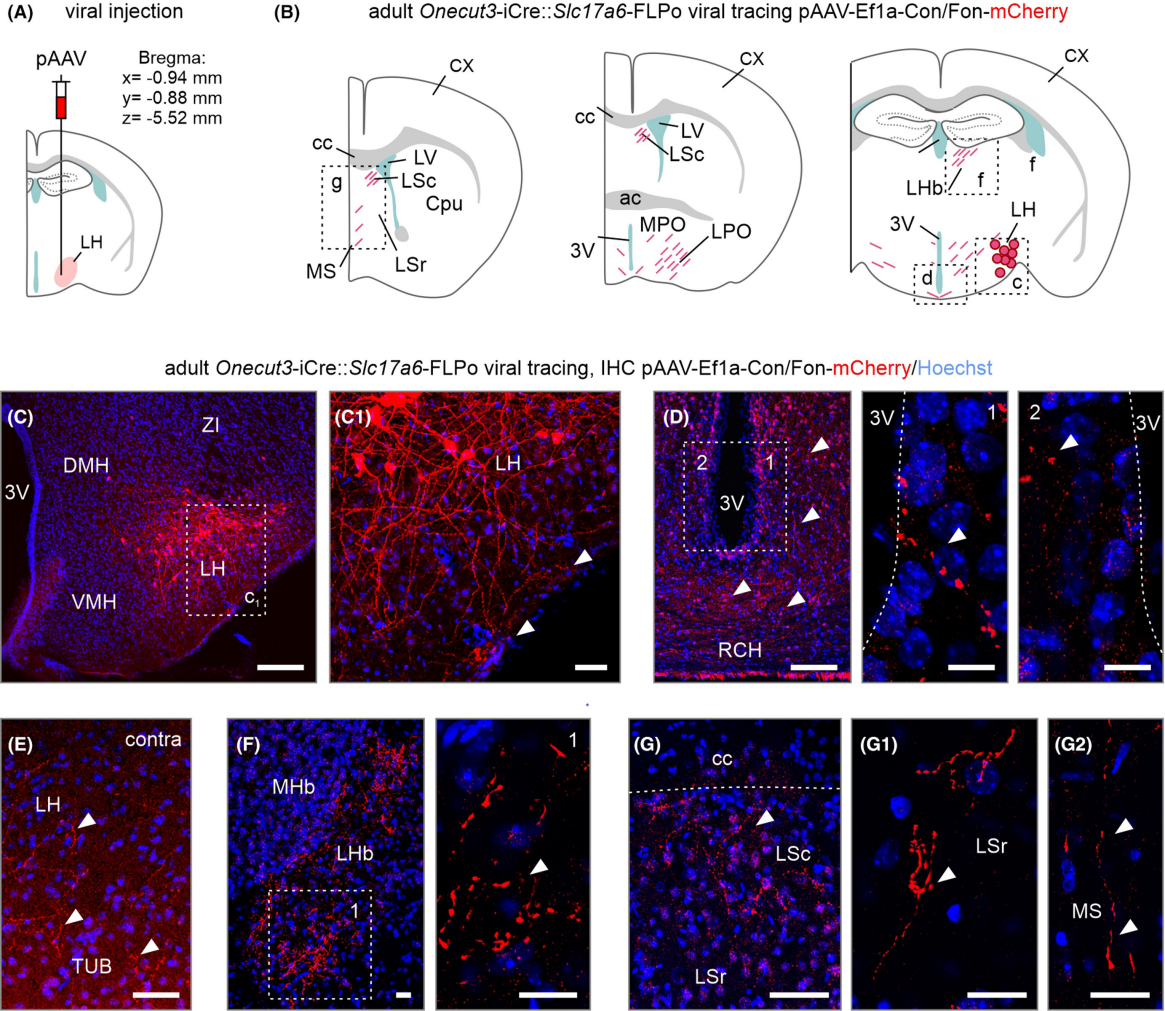
Intersectional genetic tracing of the efferent projections from *Onecut3* ^+^/*Slc17a6* ^+^ neurons in the lateral hypothalamus. (A) Coordinates relative to bregma used for virus delivery in the lateral hypothalamus (LH). (B) A dual Cre‐ and FLP‐dependent mCherry virus (pAAV‐Ef1a‐Con/Fon‐mCherry) was used to transduce *Slc17a6* ^+^/*Onecut3* ^+^ neurons. (C, C_1_) Injection site in the lateral hypothalamus (LH). Arrowheads point to processes, likely dendrites, coursing along the pial surface. (D) Small‐caliber fibers with pearl‐lace‐like varicosities, likely axons, concentrated around the 3rd ventricle (3V) and throughout the pial surface of the retrochiasmatic area (RCH). (E) *Arrowheads* point to local axon collaterals in the LH and tuberal nucleus (TUB). (F) Axons were also present in the lateral (LHb), but not medial habenula (MHb). (G, G_1_) mCherry^+^ processes were located in both the rostral and caudal lateral septal nuclei (LSc/LSr), as well as the medial septum (MS; G_2_). 3V, 3rd ventricle; cc, corpus callosum; Cpu, caudate putamen; DMH, dorsomedial hypothalamus; LPO, lateral preoptic area; LV, lateral ventricle; MPO, medial preoptic area; RCH, retrochiasmatic area; VMH, ventromedial hypothalamus. Scale bars = 500 μm (C), 250 μm (D, F, G), 50 μm (C_1_), 20 μm (G_1_, G_2_), 10 μm (D inset; F inset).

**FIGURE 6:**
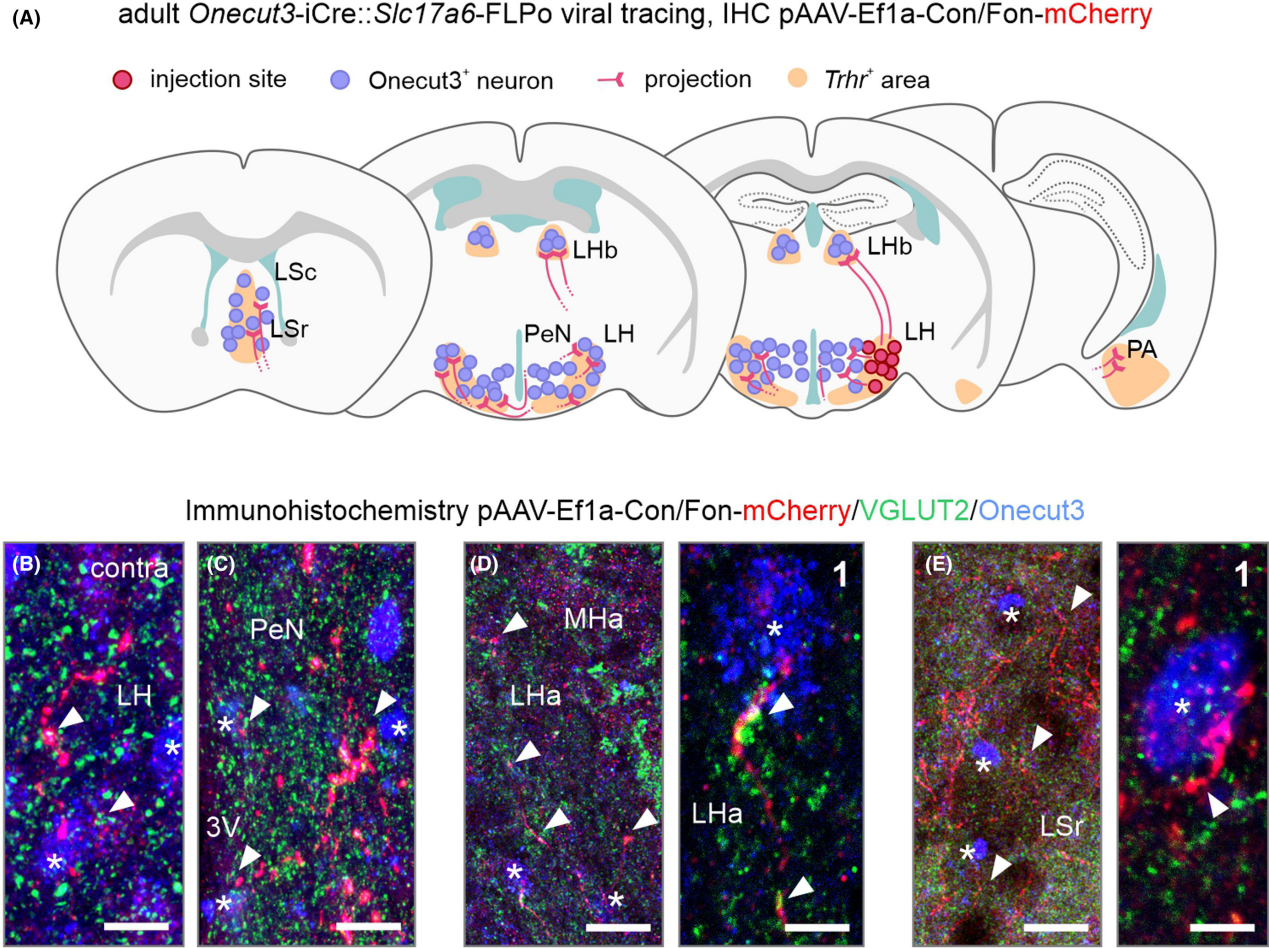
Global connectivity of lateral hypothalamic *Onecut3/Vglut2* ^+^ projections. (A) Schematic representative map of mCherry traced projections (red). Note the termination of axons in brain regions containing both *Onecut3* ^*+*^ neurons (blue), as well as strong *Trh* receptor (*Trhr*) expression (orange). (B–E) Termination fields of mCherry positive fibers (red, arrowheads) interspersed with *Onecut3* ^*+*^ nuclei (blue, asterisk) in the contralateral lateral hypothalamus (B), periventricular nucleus (C), habenula complex (D) and lateral septum (E). Note the presence of VGLUT2 protein (green) in mCherry^+^ varicosities denoting synaptic structures. 3V, 3rd ventricle; Lha, lateral habenula; LSc, lateral septal nucleus caudal; LSr, lateral septal nucleus rostral; MHa, medial habenula; PA, posterior cortical nucleus of the amygdala; PeVN, periventricular nucleus. Scale bars = 30 μm (D, E), 20 μm (C), 10 μm (B, D inset, E inset).

To this end, we crossed *Onecut3*‐iCre and *Slc17a6*‐FLPo mice (the latter expressing flippase under the *Vglut2* promoter; *n* = 4; adult), and microinjected a dual Cre/FLP‐dependent pAAV‐Ef1a‐Con/Fon‐mCherry virus,^12^ expressing mCherry under the *Ef1a* promoter (Figure 5A). Thus, we restricted viral labeling to neurons co‐expressing *Slc17a6* and *Onecut3*. Alike above, we found mCherry^+^ large‐caliber varicose dendrites coursing in the lateral hypothalamus and the tuberal area, often even reaching the pial surface (Figure 5B–C_1_). Small caliber varicose fibers, likely axons, accumulated around the 3rd ventricle, including its contralateral surface by traversing through the basal retrochiasmatic area directly underneath the 3rd ventricle (Figure 5D), but avoiding the median eminence. Most unexpectedly, we also found mCherry^+^ efferents in the contralateral lateral hypothalamus and tuberal area (Figure 5E). Besides, our intersectional approach recapitulated data on terminal fields in the LHb (Figure 5F), the lateral and medial septal nuclei (Figure 5G‐G_2_), and posterior cortical nucleus of the amygdala (Figure S5A,A_1_). However, these efferents were restricted unilaterally within the injected hemisphere. By using an intersectional approach, we did not recapitulate the fiber labeling in the hippocampus and cortex, indicating that these fibers are unlikely to be of *Onecut3* ^*+*^ */Vglut2* ^*+*^ origin (Figure S5B,C). Similar to the tracing above (Figure 4), we found projections to regions containing *Onecut3* ^+^ neurons **(**Figure 6A). Axonal projections were intermingled between *Onecut3* ^+^ territories in the lateral hypothalamus/tuberal region, periventricular nucleus, and preoptic area on both sides, as well as the ipsilateral medial and lateral septum, and LHb (Figure 6B). Immunohistochemistry confirmed the presence of VGLUT2 in varicosities throughout the projections, as well as in the vicinity of *Onecut3* ^+^ neurons, suggesting direct glutamatergic innervation (Figure 6B–E).

By using previously published hypothalamic single cell mRNA datasets,^1^, ^2^ we found that the cellular identity of *Onecut3* ^*+*^ */Vglut2* ^*+*^ neurons in the lateral hypothalamus was split: 62% co‐expressed *Trh*, with only 13.6% being non‐*Onecut3* Trh^*−*^ */Vglut2* ^*+*^ neurons (Figure S6A). These findings were confirmed by in situ hybridization (Figure S6B). Upon in‐depth analysis, we did not find *Onecut3* ^*+*^ */Trh* ^*−*^ neurons, even when considering broader neuropeptide signatures for subtype diversification. For instance, somatostatin, galanin, prodynorphin, and even brain derived neurotrophic factor (BDNF) were found in both *Trh* ^+^/*Onecut3* ^+^, and *Trh* ^−^/*Onecut3* ^+^ subgroups. *Trh* ^−^ vs. *Trh* ^+^ populations were closely matched (Figure S6C). Indeed, another detailed single cell study from the lateral hypothalamus describes 7 *Slc17a6* ^*+*^ subgroups, containing both *Onecut2* (*Onecut3* paralog expressed in in overlapping populations) and *Trh*, with no other glutamatergic neurons expressing either without the other,^28^ strongly suggesting a complete pool of glutamatergic *Onecut3* ^*+*^ */Trh* ^*+*^ containing cells. Furthermore, the major efferent projections described above were detected in regions significantly expressing the *Trh* receptor (*Trhr*), including the tuberal nucleus, lateral hypothalamus, septum, LHb, and the posterior cortical nucleus of the amygdala, indicating that most traced neurons were likely *Trh* ^*+*^ (Figure 6A; Figure S7A–D). Cumulatively, these data suggest that *Onecut3* ^+^ neurons in the lateral hypothalamus form extrahypothalamic projections towards brain areas that are *Trhr* ^+^, and associated with learning and memory, an arrangement compatible with cognitive deficits upon congenital hypothyroidism.^29^, ^30^

## 3 DISCUSSION

The understanding of neuronal complexity and connectivity in discrete brain regions had recently received major support from the introduction of single‐cell RNA‐seq technologies, allowing the revised classification of neuronal subtypes based on their RNA landscapes.^1^, ^2^, ^3^, ^4^, ^5^, ^6^ Molecular profiling, particularly over successive developmental stages, predominantly relies on the spatiotemporal expression of TFs and their gene regulatory networks instructing the acquiring of distinct neurochemical make‐ups.^1^ Derived from recent studies of ourselves,^1^, ^2^ as well as open‐label datasets,^4^ the peculiar case of the *Onecut3* TF was selected, because it is retained during postnatal development and even in adulthood. This feature could suggest novel functions likely related to the maintenance of synaptic neurotransmission and/or connectivity and cellular plasticity, as seen for Sox and POU TFs in the cerebral cortex.^31^, ^32^, ^33^ Nevertheless, a caveat in existing knowledge on the precise neurocircuit established by *Onecut3* ^+^ neurons precludes correct inferences on postnatal functions.

To overcome these limitations, we have produced transgenic mice that allow for either fluorescent reporters or iCre recombinase be expressed under the control of regulatory elements of the *Onecut3* promoter. By using our transgenic lines, we present the detailed mapping of the *Onecut3* connectome in both the fetal and adult brains. Besides our immediate aims, the combination of *Onecut3*‐iCre mice with the viral delivery of either optogenetic^34^ or DREADD constructs^35^ for the temporally restricted control of neuronal activity presents powerful means to study the functionality of *Onecut3* ^+^ neurons in intact systems in vivo in the future. Likewise, the manipulation of select genes (or gene sets) in *Onecut3* ^+^ neurons by, e.g., Cre‐dependent shRNA expression^36^ can provide information pertinent to the regulation of neuronal output. Here, we have not only quality‐controlled these new mouse models but also provided substantial novel insights in the neurochemical heterogeneity, morphology, and connectivity of a novel subset of *Onecut3* ^*+*^/*Slc17a6* ^+^ neurons in the lateral hypothalamus, of which many are TRH^+^.

The tri‐peptide TRH was historically isolated from the hypothalamic median eminence, where it is released into the bloodstream from release terminals of neuroendocrine cells of the paraventricular nucleus^37^, ^38^ to instruct the pituitary gland. However, TRH signaling seems equally important in many other extrahypothalamic regions,^39^, ^40^ rich in TRH receptor mRNA (*Trhr*) and protein.^41^ For instance, TRH modifies action potential waveforms in cortical pyramidal cells and also modulates their acetylcholine‐induced excitation.^42^ However, previous anatomical studies were unable to elucidate the efferent projections that could deliver TRH to these forebrain regions, contrasting, for example, the well‐described glutamatergic hypocretin/orexin neurons in the same area^43^, ^44^ that gives rise to ascending projections to wide cortical and subcortical regions.^45^ Considering the distribution of virally‐labeled efferents, we suggest that the lateral hypothalamus can directly contribute to TRH signaling, likely modulating cortical network activity.

For *Onecut3* ^+^ neurons, their GABA contingent in the PeVN has recently received significant attention.^1^, ^23^ However, the excitatory subtypes in the lateral hypothalamus has not been studied in detail. Here, we describe an alternatively produced glutamatergic *Onecut3* ^+^ neuronal subtype. Major findings of our work include that (i) *Slc17a6* ^+^ */Onecut3* ^+^ neurons acquire their final positions by mid‐gestation in the mouse. This suggest that the wiring of their neurocircuits is an early event and might have a significant role in the activity‐dependent development of their postsynaptic targets.^28^ (ii) *Slc17a6* ^+^ */Onecut3* ^+^ neurons of the lateral hypothalamus innervate midline structures that are endowed with other *Onecut3* ^+^ neurons. (iii) The assembly of spatially segregated extrahypothalamic *Onecut3* ^+^ neuronal populations, if synaptically connected, could suggest a reinforcement loop to coordinate the output of phenotypically similar neurons across fore‐, mid‐, and hindbrain areas. While these *Onecut3* ^+^ regions do not share the same function per se, future studies on functional connectivity might be well‐placed to elucidate how these brain areas are interconnected to synchronize outputs for complex behaviors. Even more so, mutual innervation of the lateral hypothalamus in the contralateral hemisphere (while other targets are strictly stereotypically mapped) suggests precise brain‐wide coordination of network output. (iv) The selectivity of circuit components suggests that behavioral outputs, if any, could include reward, reactivity to adverse stimuli through fear and anxiety,^46^, ^47^, ^48^ and circadian synchronization of TRH with synaptic output (e.g., for motivational aspects including sexual behavior)^49^, ^50^, ^51^ through the recruitment of the LHb and the septal complex, as well as other *Onecut3* ^−^ structures including the cortical amygdala complex.^52^ Thus, our study identifies the efferent connectivity of *Onecut3* ^+^ neurons in the lateral hypothalamus as a prototypic blueprint for the wiring of other hypothalamic neurons, with their integration into extrahypothalamic circuitries to link neuropeptide action on neurocircuits driving motivational aspects of cognition.

## 4 MATERIALS AND METHODS

Mice were housed in a temperature‐controlled environment with a 12‐h/12‐h dark‐–light cycle, and free access to food and water. All experimental procedures were planned to reduce suffering, as well as animal numbers. Transgenic lines used in this study were listed in Table 1.

### 4.1 Generation of transgenic mice

Mice expressing either mCherry or iCre recombinase under the control of regulatory elements of the *Onecut3* promoter were generated using a BAC‐based approach. (BAC)*Onecut3*‐mCherry and (BAC)*Onecut3*‐iCre mice were custom designed, ES cells injected on an FVB background, and backcrossed onto the C57Bl/6J background for >5 generations before use (Table 1). BAC clones RP23‐161H22 (Source BioScience) and B6Ng01‐345A14 (RIKEN BioResource Research Center) were used for (BAC)*Onecut3* lines. Modified BAC cassettes contained cDNA of either mCherry or iCre recombinase, Woodchuck hepatitis virus posttranscriptional regulatory elements (WPRE), human growth hormone polyadenylation hormone (hGH‐PA), and a neomycin selection cassette flanked by flippase recognition sites. The transgene was fused into the translation initiation codon (ATG site) of the *Onecut3* gene. The *Onecut3*‐mCherry construct additionally included Sleeping Beauty inverted repeats that were designed to increase integration efficiency. The neomycin cassette was excised through FLP‐mediated recombination, and the isolated transgenic fragment was microinjected into the pronucleus of fertilized mouse eggs (FVB.129P2‐Pde6b^+^Tyrc‐ch/AntJ, Jackson Laboratory, stock number #004828). Mice were genotyped by standard genotyping (Table 2) to confirm transgene expression.

### 4.2 Genotyping

DNA was extracted from tail (embryos) or toe clips (postnatal pups) using a standard lysis protocols with 50 mM NaOH solution (Sigma). Genotypes were subsequently analyzed with AccuStart II PCR SuperMix (Avantor/VWR) and appropriate primer pairs (Table 2) by using a BioRad thermocycler T100.

### 4.3 Tissue collection and fixation

Timed pregnancies were produced by housing a male with one or two female mice and detecting a vaginal plug the morning after intercourse (designated as embryonic [E] day 0.5). Whole embryos (up to stage E12.5), whole heads of embryos (E14.5) or dissected brains (P3) were collected, and immersion fixed with 4% paraformaldehyde (PFA) in 0.1 M PB (pH 7.4) at 4°C for 2‐24 h under continuous agitation. For older postnatal and adult brains, mice were transcardially perfused with a 4% PFA in 0.1 M PB (pH 7.4) and post‐fixed in the same solution overnight (at 4°C with agitation). Samples were cryoprotected in 30% sucrose in 0.1 M PB for >2 days prior to cryosectioning.

### 4.4 Quantitative polymerase chain reactio

Total RNA was extracted from brain tissue samples using the Aurum Total RNA kit (BioRad), and reverse transcribed into cDNA with a High‐Capacity RNA‐to‐cDNA Kit (Applied Biosystems). A total of 5–20 ng of cDNA was used for quantitative real‐time PCR (CFX Connect, BioRad) when mixed with a SYBR Green Master Mix Kit (Life Technologies). Primers were custom designed (Primer Blast; National Center for Biotechnology) and listed in Table 3. Expression levels were normalized to TATA box‐binding protein (*Tbp*), a housekeeping gene.

**TABLE 3:** qPCR primers*.*

Target gene	Primer pairs
*Onecut3*	Forward: 5′‐GCTGATTGCCATCTTCAAGG‐3′ Reverse: 5′‐ GAAGTTGCTGACAGTGTTGA‐3′
*Tbp*	Forward: 5′‐CCTTGTACCCTTCACCAATGAC‐3′ Reverse: 5′‐ACAGCCAAGATTCACGGTAGA‐3′

### 4.5 In situ hybridization

Fresh‐frozen brains were sectioned (16 μm thickness) on a CryoStar NX70 cryostat microtome and collected on SuperFrost^+^ glass slides (ThermoFisher). Sections were immersed in 4% PFA solution for 20 min, followed by repeated washes with phosphate‐buffered saline (0.05 M, pH 7.4), and dehydration in an ascending ethanol gradient (25%, 50%, 75%, and 100%; 5 min each). The HCR c3.0 protocol (Molecular Instruments) was used for in situ hybridization with the *Onecut3*, *Trh* and *Slc17a6* probes. Samples were imaged on an LSM 880 confocal microscope (Zeiss) at 40× or 63× primary magnification.

### 4.6 Fluorescence immunohistochemistry

Immunohistochemistry was performed on 20‐μm thick cryosections (for ages up to P3) or 50‐μm thick free‐floating sections. Sections were washed with 0.05 M PBS and incubated with a blocking solution containing 5% normal donkey serum (NDS, Jackson ImmunoResearch), 2% bovine serum albumin (BSA, Sigma), 0.2% Triton X‐100 (Sigma) in PBS (22–24 °C, 1 h). Next, tissues were exposed to a solution containing 2% NDS, 0.1% BSA, 0.2% Triton X‐100 in PBS and a pre‐defined mixtures of primary antibodies (Table 4) at 4°C for 72 h. After extensive rinsing in 0.05 M PBS, secondary antibodies conjugated to cyanine (Cy)2, Cy3.,or Cy5 (1:300, made in donkey; Jackson ImmunoResearch) were applied (22–24°C, 2 h). Hoechst 33342 (1:10000, Sigma, #23491‐52‐3) was routinely used as a nuclear counterstain. After repeated washes in PBS, sections were dipped in distilled water, mounted, air‐dried, and coverslipped with Entellan (in toluene; Merck).

**TABLE 4:** Antibody list.

Antibody	Host species	Concentration	Manufacturer
Onecut3	Guinea pig	1:5000	Provided by F. Clotman^53^
CPCA‐mCherry	Chicken	1:1000	EnCor #CPCA‐mCherry
GFP‐FITC	Goat	1:1000	Abcam #ab6662
VGLUT2	Rabbit	1:1000	Synaptic Systems
Parvalbumin	Rabbit	1:2500	Swant
SMI‐32	Mouse	1:1000	Sternberger
Hoechst 33342	—	1.10000	Sigma #14533

### 4.7 Whole‐mount immunofluorescence

E9.5 and E10.5 mouse embryos were immersion fixed (4% PFA in 0.1 M PB) at 4°C for 2 h, followed by repeated washes in PBS‐Tween (0.1% Tween‐20 in 0.05 M PBS). Embryos were then immersed in increasing concentrations of methanol (25%, 50%, 75%, 100% for 1 h each) and bleached overnight in a solution containing 1 part 30% H_2_O_2_ and 2 parts Dent's fixative (20% dimethyl sulfoxide and 80% methanol). Samples were then washed with 100% methanol and immersed in Dent's fix at 4°C for 24 h, followed by incubation in a mixture of 5% NDS, 20% DSMO, 75% PBS‐Tween and appropriate combinations of primary antibodies (Table 4) in glass vials for 7 days (at 22–24°C, under continuous rotation), followed by 3‐day incubation (22–24°C) with secondary antibodies. Samples were washed with PBS‐Tween (6× 30 min), 50% methanol/PBS (5 min), and 100% methanol (3× 20 min). Finally, samples were cleared in BABB (1 part benzyl alcohol and 2 parts benzyl benzoate) and imaged with a LSM 880 confocal laser scanning microscope (Zeiss). Whole‐embryo 3D reconstructions were in ZEN software (Zeiss) by using the orthogonal stacking and tile‐scan modes, and processed in Imaris (X64 9.0.2, Bitplane).

### 4.8 AAV‐based tracing in vivo

Adult mice were anesthetized with an *i.p*. injection of ketamine (90 mg/kg) and xylazine (10 mg/kg) and their heads placed in a stereotaxic frame (Kopf instruments). Mice were kept anesthetized by inhalation of N_2_O/isoflurane (0.5%–1%, 1 L/min flow rate). Each mouse received an injection of 30 μL meloxicam (2 mg/mL; given *s.c*.) and 50 μL enrofloxacin (25 mg/mL; given *s.c*.) prior to the surgical procedure. The skull was exposed, and a burr‐hole craniotomy performed to make the brain at the AP = −0.94 mm, L = −0.88 mm, and DV = 5.52 mm coordinates (relative to bregma). A stereotaxic injector connected to a capillary made of borosilicate glass was used to infuse 200 nL of viral particles (see Table 5 for a complete list of virus constructs) in 1 min. After 5 min, the glass capillary was slowly withdrawn. The incision site was stitched, and the mice kept under an infrared lamp to aid their recovery, and closely monitored until they have regained consciousness. Mice were sacrificed 3 weeks later and processed for immunohistochemistry as earlier.

**TABLE 5:** List of AAV virus constructs.

ID	Name	Manufacturer
AAV‐168	FlpOn‐GFP	Per Wulff
50 459‐AAV8	pAAV‐hSyn‐DIO‐mCherry	Addgene
137 132‐AAV8	pAAV‐Ef1a‐Con/Fon‐mCherry	Addgene

## FUNDING INFORMATION

This work was supported by the Austrian Science Fund (FWF, P 34121‐B; E.K), the Swedish Research Council (2018‐02838, T.Ha.; 2020‐01688, T.Hö.), the Swedish Brain Foundation (Hjärnfonden, FO2022‐0300, T.Ha.), the Novo Nordisk Foundation (NNF20OC0053667, T.Ha.), the European Research Council (FOODFORLIFE, ERC‐2020‐AdG‐101 021 016; T.Ha), and the Arvid Carlsson Foundation (T.Hö).

## CONFLICT OF INTEREST STATEMENT

T.Hö. declares stocks in Lundbeck and Bioarctic. Other authors of this manuscript declare no conflict of interest. Data are available upon request from the authors.

## Supporting information


Figure S1.

Figure S2.

Figure S3.

Figure S4.

Figure S5.

Figure S6.

Figure S7.

## References

[1] Romanov RA , Tretiakov EO , Kastriti ME , et al. Molecular design of hypothalamus development. Nature. 2020;1–7:246‐252. doi:10.1038/s41586-020-2266-0 PMC7292733 32499648

[2] Romanov RA , Zeisel A , Bakker J , et al. Molecular interrogation of hypothalamic organization reveals distinct dopamine neuronal subtypes. Nat Neurosci. 2017;20:176‐188. 27991900 10.1038/nn.4462 PMC7615022

[3] Shimogori T , Lee DA , Miranda‐Angulo A , et al. A genomic atlas of mouse hypothalamic development. Nat Neurosci. 2010;13:767‐775. 20436479 10.1038/nn.2545 PMC4067769

[4] Kim DW , Washington PW , Wang ZQ , et al. The cellular and molecular landscape of hypothalamic patterning and differentiation from embryonic to late postnatal development. Nat Commun. 2020;11:4360. 32868762 10.1038/s41467-020-18231-z PMC7459115

[5] Schredelseker T , Driever W . Conserved Genoarchitecture of the basal hypothalamus in zebrafish embryos. Front Neuroanat. 2020;14:3. 32116574 10.3389/fnana.2020.00003 PMC7016197

[6] Affinati AH , Sabatini PV , True C , et al. Cross‐species analysis defines the conservation of anatomically segregated VMH neuron populations. elife. 2021;10:e69065. 34018926 10.7554/eLife.69065 PMC8184210

[7] Lemaigre FP , Durviaux SM , Truong O , Lannoy VJ , Hsuan JJ , Rousseau GG . Hepatocyte nuclear factor 6, a transcription factor that contains a novel type of homeodomain and a single cut domain. Proc Natl Acad Sci USA. 1996;93:9460‐9464. 8790352 10.1073/pnas.93.18.9460 PMC38450

[8] Lannoy VJ , Bürglin TR , Rousseau GG , Lemaigre FP . Isoforms of hepatocyte nuclear Factor‐6 differ in DNA‐binding properties, contain a bifunctional homeodomain, and define the new ONECUT class of homeodomain proteins*. J Biol Chem. 1998;273:13552‐13562. 9593691 10.1074/jbc.273.22.13552

[9] Iyaguchi D , Yao M , Watanabe N , Nishihira J , Tanaka I . DNA recognition mechanism of the ONECUT homeodomain of transcription factor HNF‐6. Structure. 2007;15:75‐83. 17223534 10.1016/j.str.2006.11.004

[10] Zhang Y‐H , Xu M , Shi X , et al. Cascade diversification directs generation of neuronal diversity in the hypothalamus. Cell Stem Cell. 2021;28:1483‐1499.e8. 33887179 10.1016/j.stem.2021.03.020

[11] Heintz N . Bac to the future: the use of bac transgenic mice for neuroscience research. Nat Rev Neurosci. 2001;2:861‐870. 11733793 10.1038/35104049

[12] Fenno LE , Ramakrishnan C , Kim YS , et al. Comprehensive dual‐ and triple‐feature intersectional single‐vector delivery of diverse functional payloads to cells of behaving mammals. Neuron. 2020;107:836‐853.e11. 32574559 10.1016/j.neuron.2020.06.003 PMC7687746

[13] Vanhorenbeeck V , Jenny M , Cornut JF , et al. Role of the Onecut transcription factors in pancreas morphogenesis and in pancreatic and enteric endocrine differentiation. Dev Biol. 2007;305:685‐694. 17400205 10.1016/j.ydbio.2007.02.027

[14] Roy A , Francius C , Rousso DL , et al. Onecut transcription factors act upstream of Isl1 to regulate spinal motoneuron diversification. Development. 2012;139:3109‐3119. 22833130 10.1242/dev.078501 PMC4074251

[15] Kabayiza KU , Masgutova G , Harris A , Rucchin V , Jacob B , Clotman F . The Onecut transcription factors regulate differentiation and distribution of dorsal interneurons during spinal cord development. Front Mol Neurosci. 2017;10. 10.3389/fnmol.2017.00157 PMC5445119 28603487

[16] Harris A , Masgutova G , Collin A , et al. Onecut factors and Pou2f2 regulate the distribution of V2 interneurons in the mouse developing spinal cord. Front Cell Neurosci. 2019;13. 10.3389/fncel.2019.00184 PMC6561314 31231191

[17] Toch M , Clotman F . CBP and p300 coactivators contribute to the maintenance of Isl1 expression by the Onecut transcription factors in embryonic spinal motor neurons. Mol Cell Neurosci. 2019;101:103411. 31648029 10.1016/j.mcn.2019.103411

[18] Toch M , Harris A , Schakman O , et al. Onecut‐dependent Nkx6.2 transcription factor expression is required for proper formation and activity of spinal locomotor circuits. Sci Rep. 2020;10:996. 31969659 10.1038/s41598-020-57945-4 PMC6976625

[19] Jacquemin P , Pierreux CE , Fierens S , van Eyll JM , Lemaigre FP , Rousseau GG . Cloning and embryonic expression pattern of the mouse Onecut transcription factor OC‐2. Gene Expr Patterns. 2003;3:639‐644. 12971999 10.1016/s1567-133x(03)00110-8

[20] Clotman F , Lannoy VJ , Reber M , et al. The onecut transcription factor HNF6 is required for normal development of the biliary tract. Development. 2002;129:1819‐1828. 11934848 10.1242/dev.129.8.1819

[21] Pierreux CE , Vanhorenbeeck V , Jacquemin P , Lemaigre FP , Rousseau GG . The transcription factor hepatocyte nuclear factor‐6/Onecut‐1 controls the expression of its paralog Onecut‐3 in developing mouse endoderm. J Biol Chem. 2004;279:51298‐51304. 15381696 10.1074/jbc.M409038200

[22] Berghuis P , Rajnicek AM , Morozov YM , et al. Hardwiring the brain: endocannabinoids shape neuronal connectivity. Science. 2007;316:1212‐1216. 17525344 10.1126/science.1137406

[23] Benowitz LI , Routtenberg A . GAP‐43: an intrinsic determinant of neuronal development and plasticity. Trends Neurosci. 1997;20:84‐91. 9023877 10.1016/s0166-2236(96)10072-2

[24] Méndez‐Maldonado K , Vega‐López GA , Aybar MJ , Velasco I . Neurogenesis from neural crest cells: molecular mechanisms in the formation of cranial nerves and ganglia. Front Cell Dev Biol. 2020;8:635. 32850790 10.3389/fcell.2020.00635 PMC7427511

[25] Sapkota D , Mu X . Onecut transcription factors in retinal development and maintenance. Neural Regen Res. 2015;10:899‐900. 26199604 10.4103/1673-5374.158350 PMC4498349

[26] Voelker CCJ , Garin N , Taylor JS , Gähwiler BH , Hornung JP , Molnár Z . Selective neurofilament (SMI‐32, FNP‐7 and N200) expression in subpopulations of layer V pyramidal neurons in vivo and in vitro. Cereb Cortex. 2004;14:1276‐1286. 15166101 10.1093/cercor/bhh089

[27] Tallini YN , Shui B , Greene KS , et al. BAC transgenic mice express enhanced green fluorescent protein in central and peripheral cholinergic neurons. Physiol Genomics. 2006;27:391‐397. 16940431 10.1152/physiolgenomics.00092.2006

[28] Wang Y , Eddison M , Fleishman G , et al. EASI‐FISH for thick tissue defines lateral hypothalamus spatio‐molecular organization. Cell. 2021;184:6361‐6377.e24. 34875226 10.1016/j.cell.2021.11.024

[29] Samuels MH . Psychiatric and cognitive manifestations of hypothyroidism. Curr Opin Endocrinol Diabetes Obes. 2014;21:377‐383. 25122491 10.1097/MED.0000000000000089 PMC4264616

[30] Khaleghzadeh‐Ahangar H , Talebi A , Mohseni‐Moghaddam P . Thyroid disorders and development of cognitive impairment: a review study. NEN. 2022;112:835‐844. 10.1159/000521650 34963121

[31] Deneris ES , Hobert O . Maintenance of postmitotic neuronal cell identity. Nat Neurosci. 2014;17:899‐907. 24929660 10.1038/nn.3731 PMC4472461

[32] Munguba H , Chattopadhyaya B , Nilsson S , et al. Postnatal Sox6 regulates synaptic function of cortical parvalbumin‐expressing neurons. J Neurosci. 2021;41:8876‐8886. 34503995 10.1523/JNEUROSCI.0021-21.2021 PMC8549537

[33] McClard CK , Kochukov MY , Herman I , et al. POU6f1 mediates neuropeptide‐dependent plasticity in the adult brain. J Neurosci. 2018;38:1443‐1461. 29305536 10.1523/JNEUROSCI.1641-17.2017 PMC5815346

[34] Deisseroth K , Hegemann P . The form and function of channelrhodopsin. Science. 2017;357:eaan5544. 28912215 10.1126/science.aan5544 PMC5723383

[35] Roth BL . DREADDs for neuroscientists. Neuron. 2016;89:683‐694. 26889809 10.1016/j.neuron.2016.01.040 PMC4759656

[36] Kühn R , Torres RM . Cre/loxP recombination system and gene targeting. Methods Mol Biol. 2002;180:175‐204. 11873650 10.1385/1-59259-178-7:175

[37] Schally AV , Redding TW , Bowers CY , Barrett JF . Isolation and properties of porcine thyrotropin‐releasing hormone. J Biol Chem. 1969;244:4077‐4088. 4979236

[38] Burgus R , Dunn TF , Desiderio D , Guillemin R . Molecular structure of the hypothalamic hypophysiotropic TRF factor of ovine origin: mass spectrometry demonstration of the PCA‐his‐pro‐NH2 sequence. C R Acad Hebd Seances Acad Sci D. 1969;269:1870‐1873. 4983502

[39] Jackson IM , Reichlin S . Thyrotropin‐releasing hormone (TRH): distribution in hypothalamic and extrahypothalamic brain tissues of mammalian and submammalian chordates. Endocrinology. 1974;95:854‐862. 4212470 10.1210/endo-95-3-854

[40] Oliver C , Eskay RL , Ben‐Jonathan N , Porter JC . Distribution and concentration of TRH in the rat brain. Endocrinology. 1974;95:540‐546. 4211932 10.1210/endo-95-2-540

[41] Heuer H , Schäfer MK‐H , O'Donnell D , Walker P , Bauer K . Expression of thyrotropin‐releasing hormone receptor 2 (TRH‐R2) in the central nervous system of rats. J Comp Neurol. 2000;428:319‐336. 11064370

[42] Braitman DJ , Auker CR , Carpenter DO . Thyrotropin‐releasing hormone has multiple actions in cortex. Brain Res. 1980;194:244‐248. 6247034 10.1016/0006-8993(80)91337-2

[43] de Lecea L , Kilduff TS , Peyron C , et al. The hypocretins: hypothalamus‐specific peptides with neuroexcitatory activity. Proc Natl Acad Sci U S A. 1998;95:322‐327. 9419374 10.1073/pnas.95.1.322 PMC18213

[44] Sakurai T , Amemiya A , Ishii M , et al. Orexins and orexin receptors: a family of hypothalamic neuropeptides and G protein‐coupled receptors that regulate feeding behavior. Cell. 1998;92:573‐585. 9491897 10.1016/s0092-8674(00)80949-6

[45] Peyron C , Tighe DK , van den Pol AN , et al. Neurons containing hypocretin (orexin) project to multiple neuronal systems. J Neurosci. 1998;18:9996‐10015. 9822755 10.1523/JNEUROSCI.18-23-09996.1998 PMC6793310

[46] Mondoloni S , Mameli M , Congiu M . Reward and aversion encoding in the lateral habenula for innate and learned behaviours. Transl Psychiatry. 2022;12:1‐8. 35013094 10.1038/s41398-021-01774-0 PMC8748902

[47] Calandreau L , Jaffard R , Desmedt A . Dissociated roles for the lateral and medial septum in elemental and contextual fear conditioning. Learn Mem. 2007;14:422‐429. 17554087 10.1101/lm.531407 PMC1896092

[48] Sparks PD , LeDoux JE . The septal complex as seen through the context of fear. In: Numan R , ed. The Behavioral Neuroscience of the Septal Region. Springer‐Verlag Publishing/Springer Nature; 2000:234‐269. doi:10.1007/978-1-4612-1302-4_10

[49] Korchynska S , Rebernik P , Pende M , et al. A hypothalamic dopamine locus for psychostimulant‐induced hyperlocomotion in mice. Nat Commun. 2022;13:5944. 36209152 10.1038/s41467-022-33584-3 PMC9547883

[50] Taylor JA , Boyd SK . Thyrotropin‐releasing hormone facilitates display of reproductive behavior and locomotor behavior in an amphibian. Horm Behav. 1991;25:128‐136. 1906046 10.1016/0018-506x(91)90046-k

[51] Ogawa S , Parhar IS . Role of habenula in social and reproductive behaviors in Fish: comparison with mammals. Front Behav Neurosci. 2022;15:818782. 35221943 10.3389/fnbeh.2021.818782 PMC8867168

[52] Yamaguchi T , Wei D , Song SC , Lim B , Tritsch NX , Lin D . Posterior amygdala regulates sexual and aggressive behaviors in male mice. Nat Neurosci. 2020;23:1111‐1124. 32719562 10.1038/s41593-020-0675-x PMC7483354

[53] Espana A , Clotman F . Onecut transcription factors are required for the second phase of development of the A13 dopaminergic nucleus in the mouse. J Comp Neurol. 2012;520:1424‐1441. 22102297 10.1002/cne.22803

