# Supplementary figures and images for: Brain‐wide mapping of efferent projections of glutamatergic (Onecut3+) neurons in the lateral mouse hypothalamus
Source: Acta Physiol (Oxf). 2023 Apr 25;238(3):e13973. doi: 10.1111/apha.13973 (PMC10909463; doi:10.1111/apha.13973)

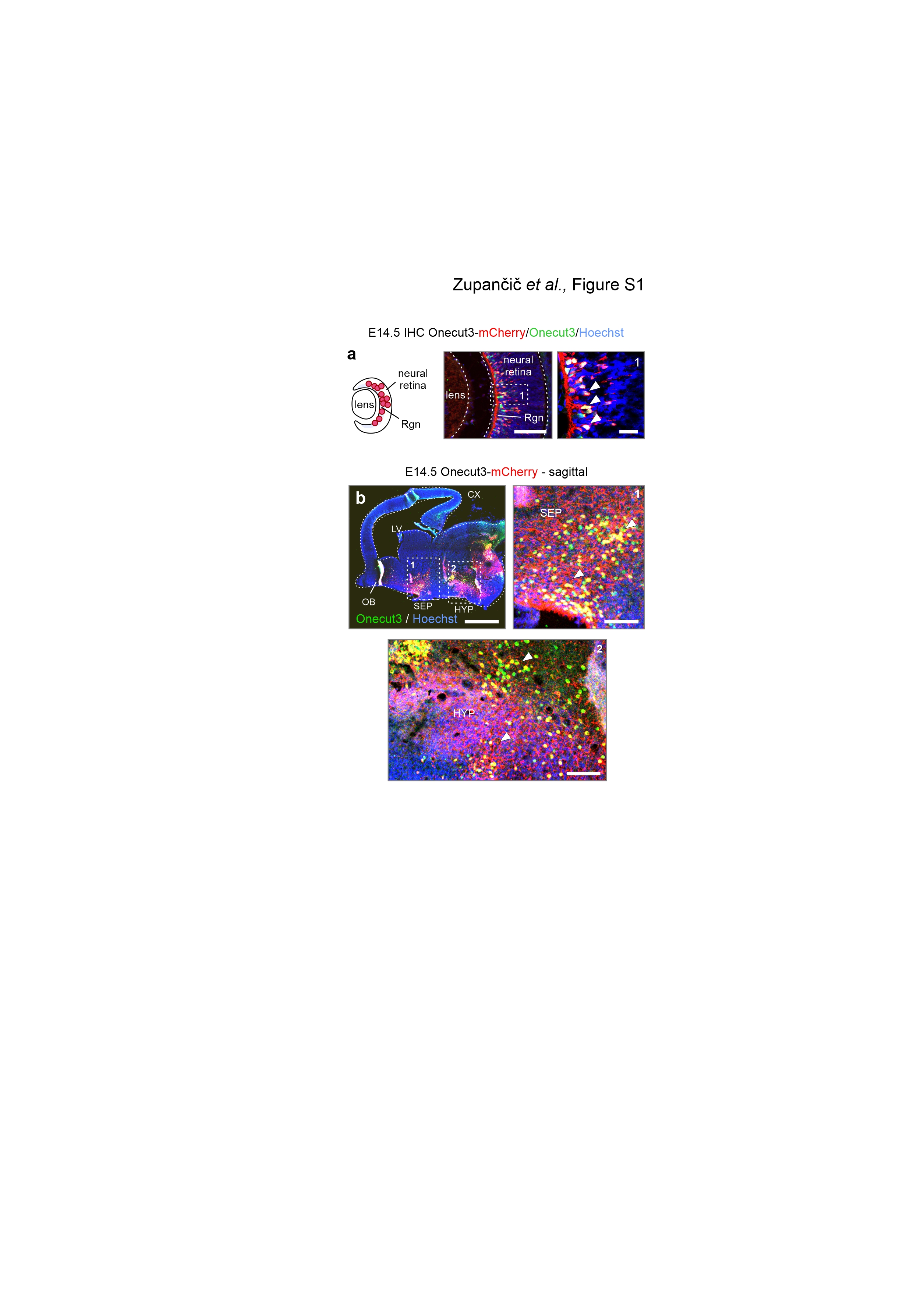

Supplement: Supplementary file 1 — Figure S1. [file APHA-238-e13973-s002.jpg]

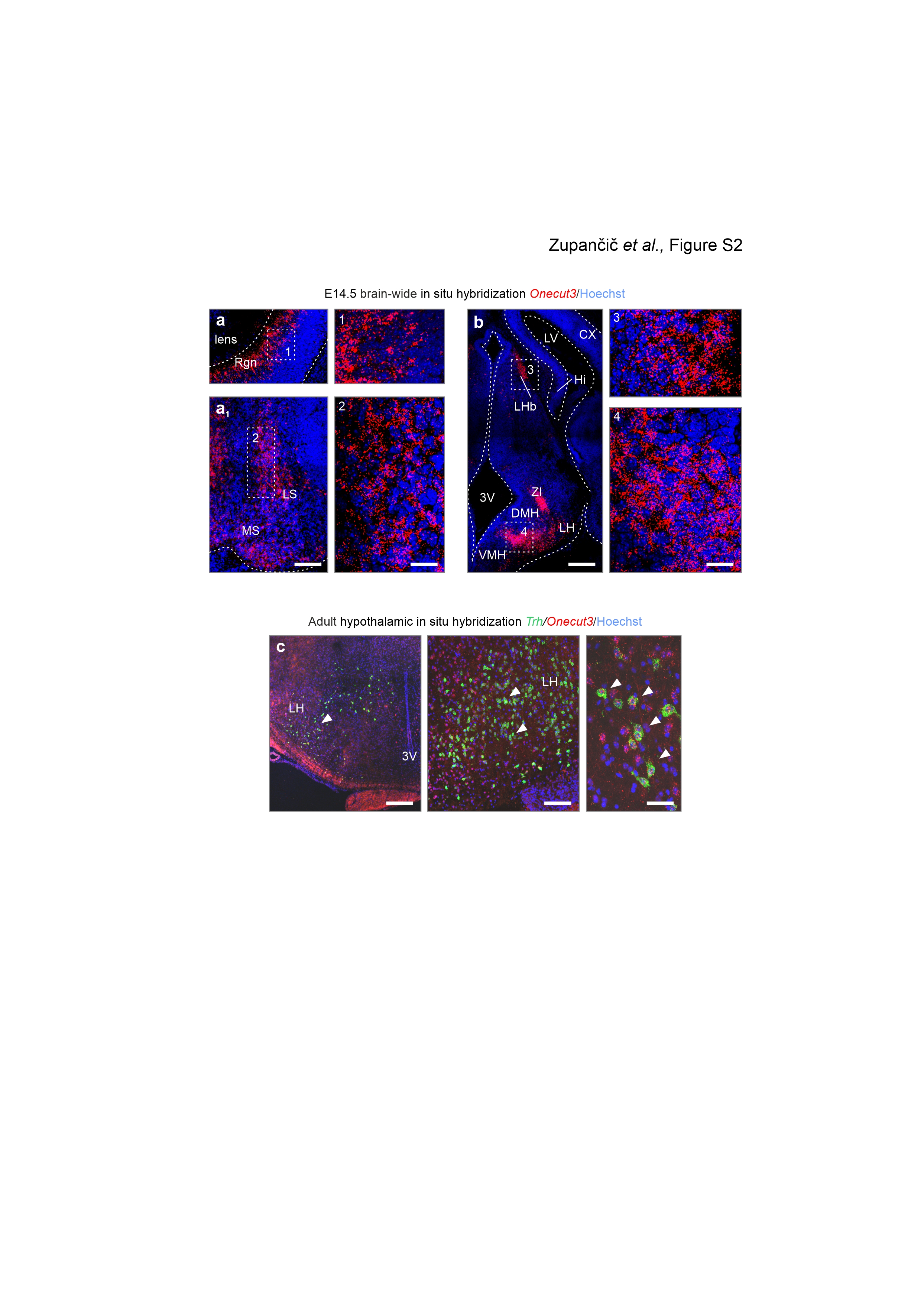

Supplement: Supplementary file 2 — Figure S2. [file APHA-238-e13973-s003.jpg]

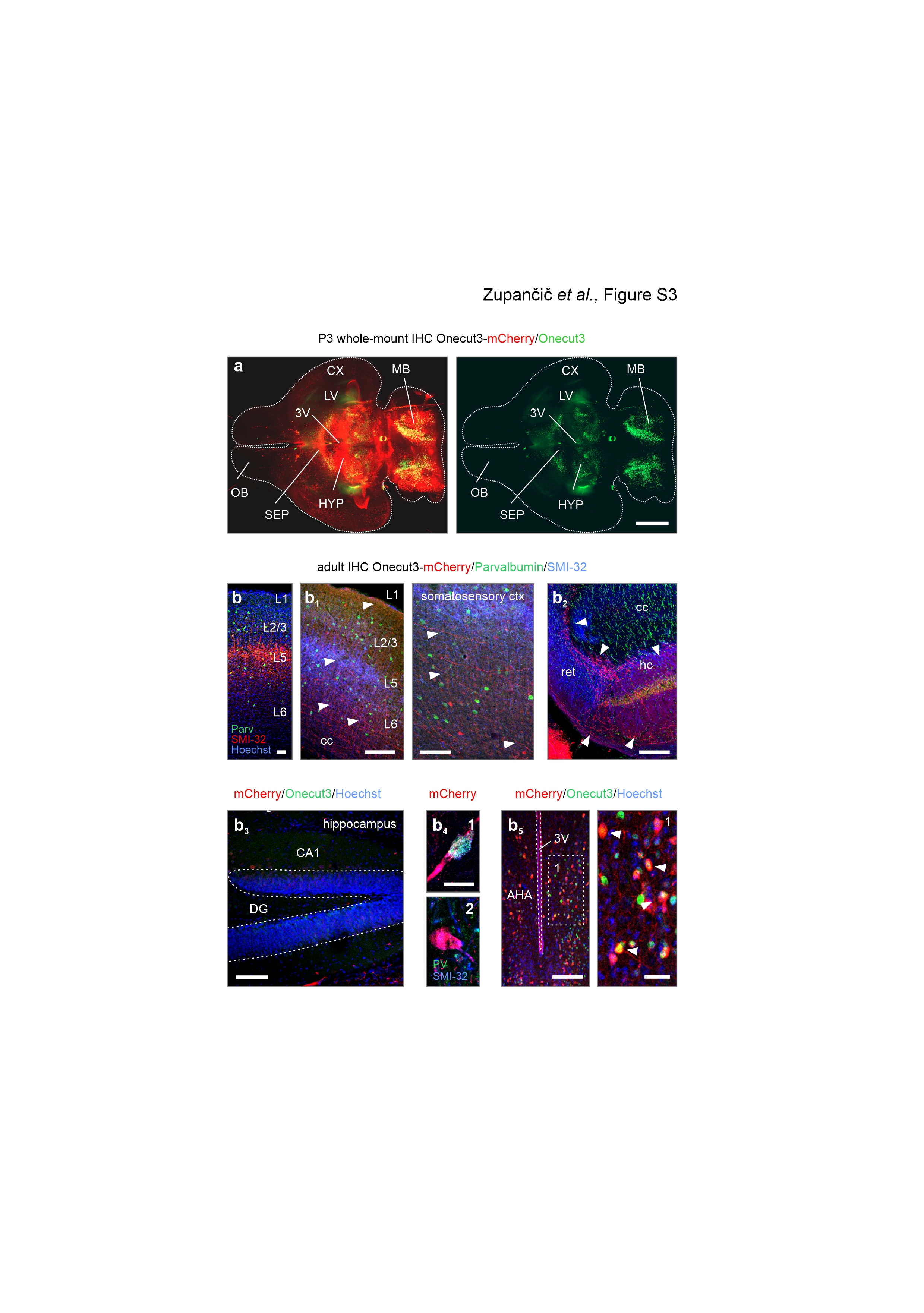

Supplement: Supplementary file 3 — Figure S3. [file APHA-238-e13973-s007.jpg]

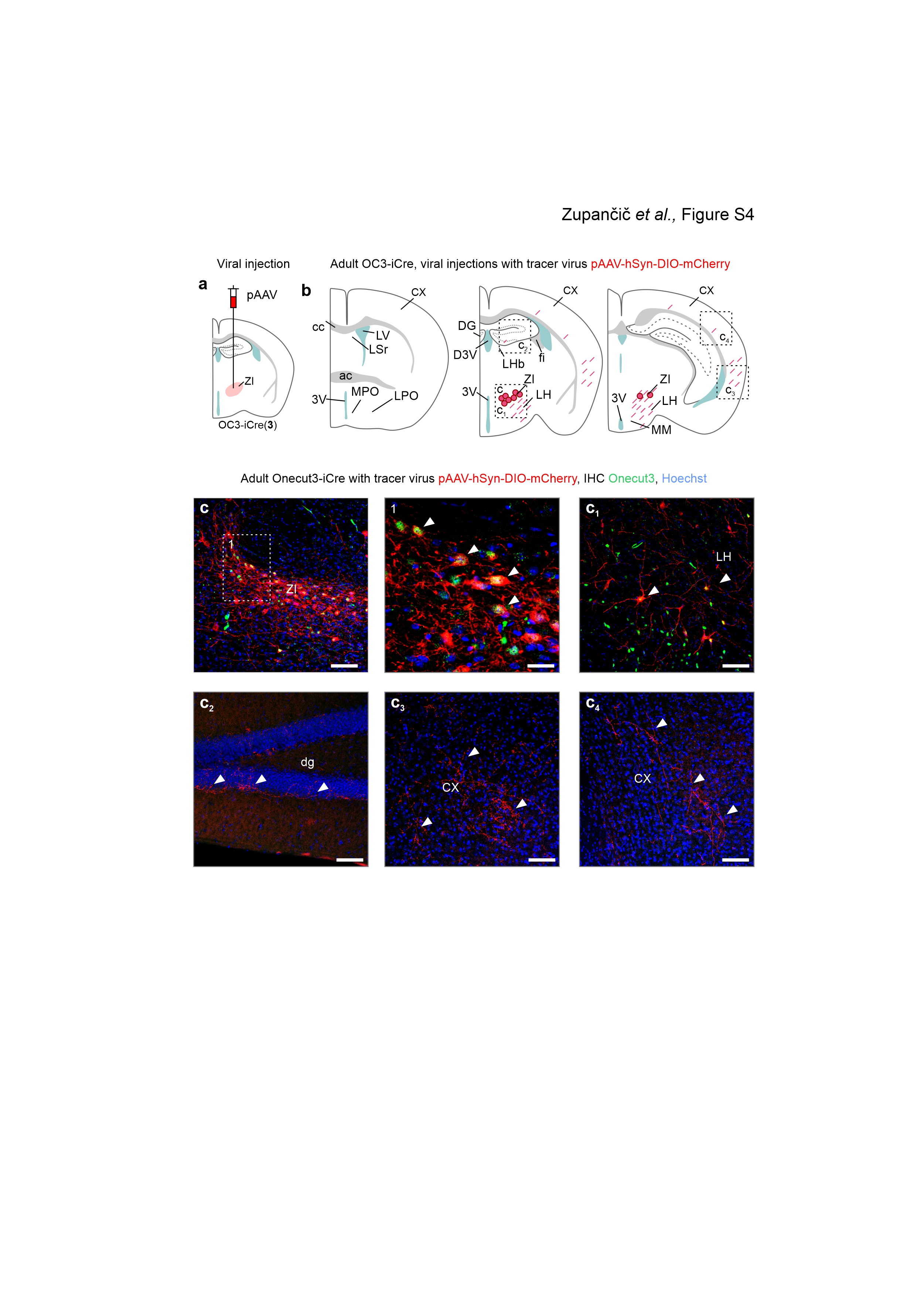

Supplement: Supplementary file 4 — Figure S4. [file APHA-238-e13973-s005.jpg]

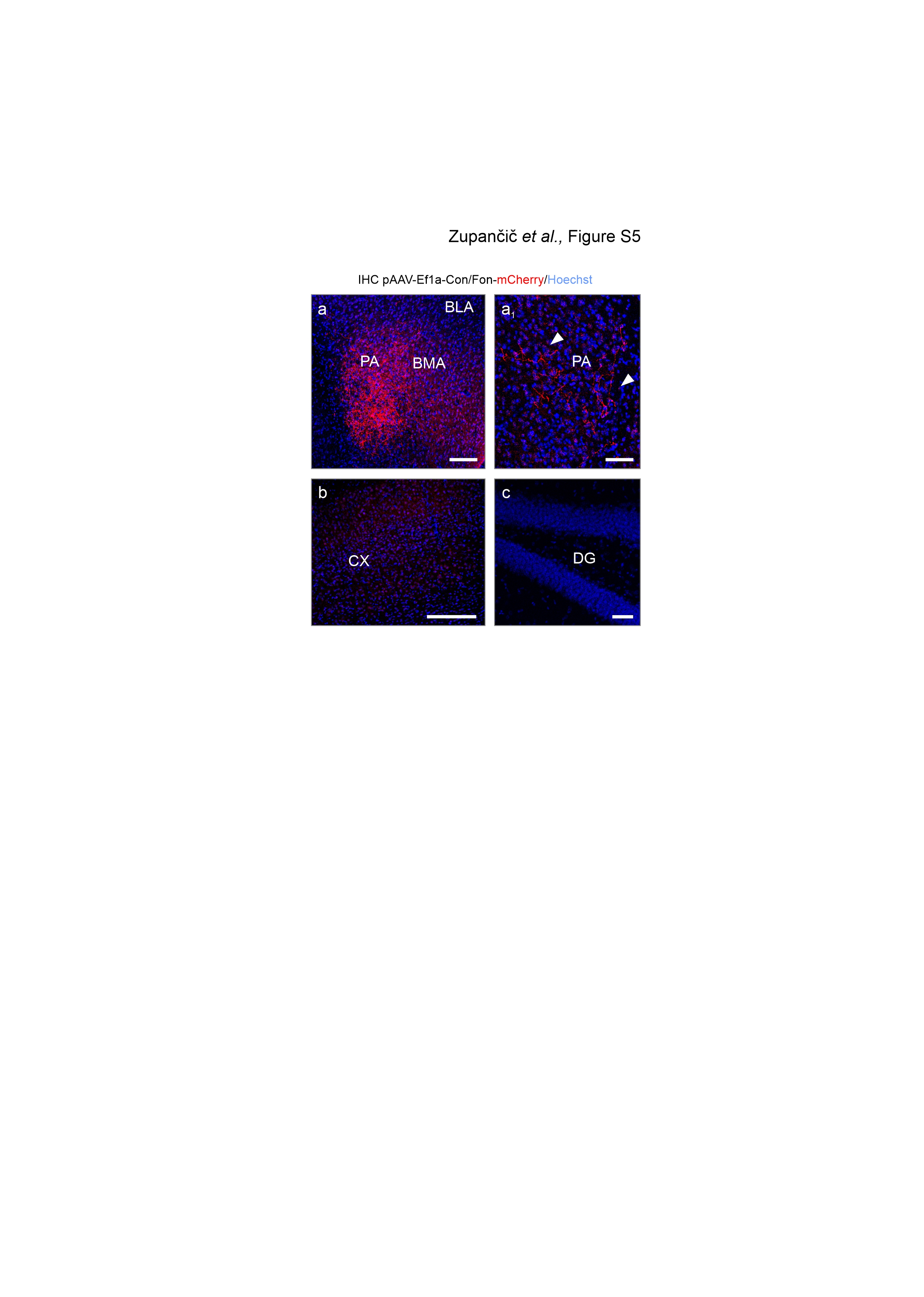

Supplement: Supplementary file 5 — Figure S5. [file APHA-238-e13973-s001.jpg]

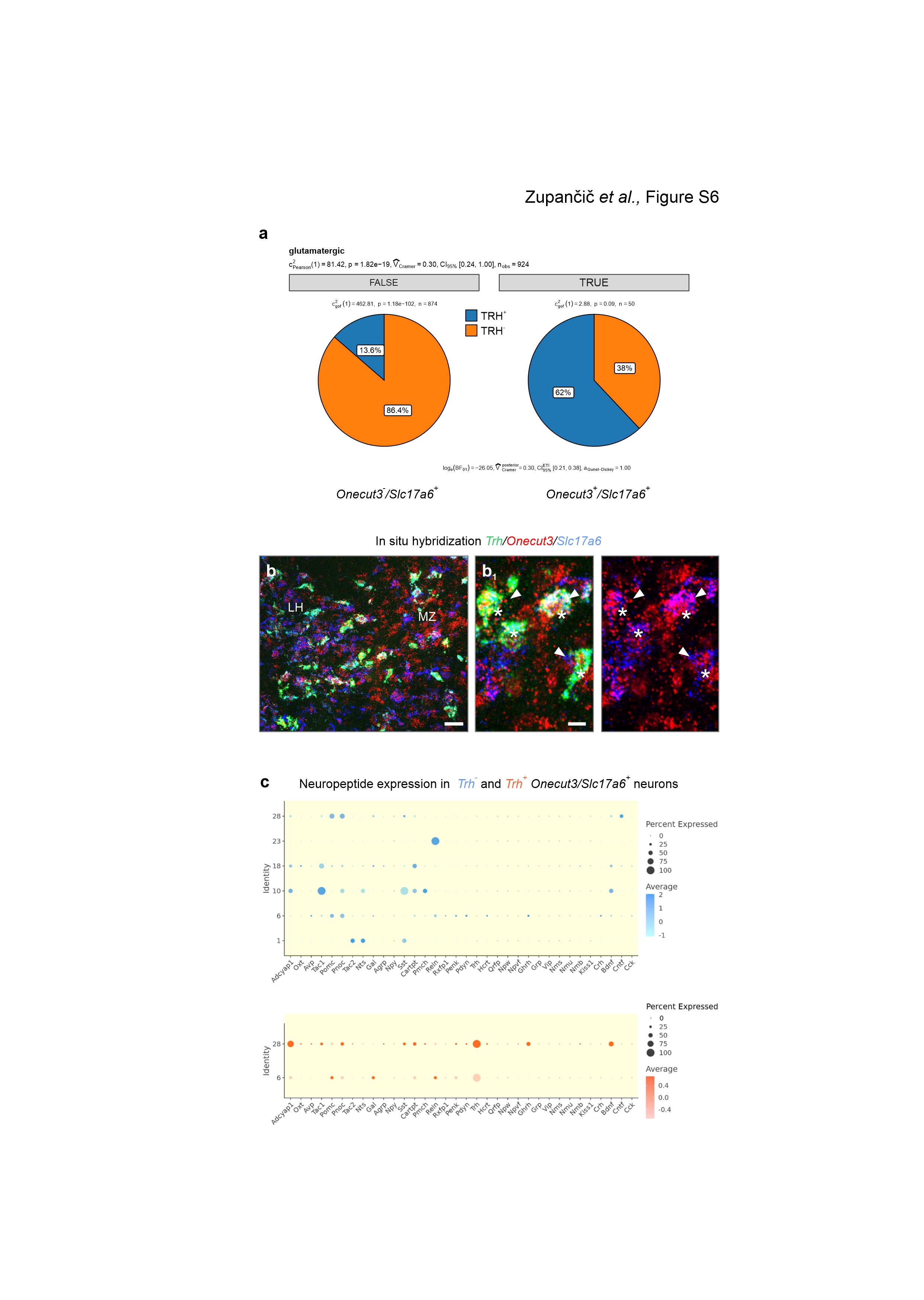

Supplement: Supplementary file 6 — Figure S6. [file APHA-238-e13973-s004.jpg]

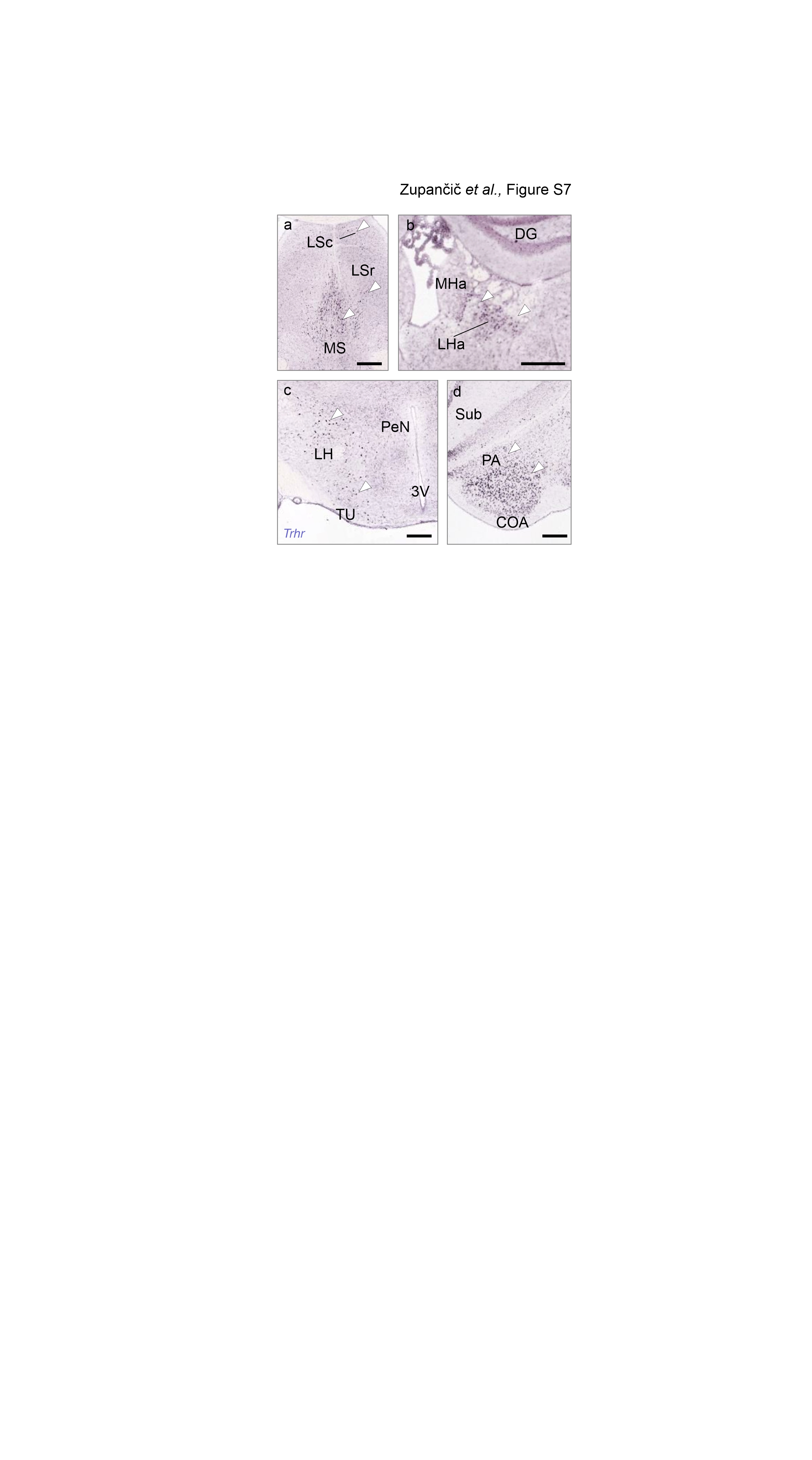

Supplement: Supplementary file 7 — Figure S7. [file APHA-238-e13973-s006.jpg]
